# Statistical properties of sketching algorithms

**DOI:** 10.1093/biomet/asaa062

**Published:** 2020-07-30

**Authors:** D. C. Ahfock, W. J. Astle, S. Richardson

**Affiliations:** MRC Biostatistics Unit, University of Cambridge, Robinson Way, Cambridge CB2 0SR, U.K.

**Keywords:** Computational efficiency, Random projection, Randomized numerical linear algebra, Sketching

## Abstract

Sketching is a probabilistic data compression technique that has been largely developed by the computer science community. Numerical operations on big datasets can be intolerably slow; sketching algorithms address this issue by generating a smaller surrogate dataset. Typically, inference proceeds on the compressed dataset. Sketching algorithms generally use random projections to compress the original dataset, and this stochastic generation process makes them amenable to statistical analysis. We argue that the sketched data can be modelled as a random sample, thus placing this family of data compression methods firmly within an inferential framework. In particular, we focus on the Gaussian, Hadamard and Clarkson–Woodruff sketches and their use in single-pass sketching algorithms for linear regression with huge samples. We explore the statistical properties of sketched regression algorithms and derive new distributional results for a large class of sketching estimators. A key result is a conditional central limit theorem for data-oblivious sketches. An important finding is that the best choice of sketching algorithm in terms of mean squared error is related to the signal-to-noise ratio in the source dataset. Finally, we demonstrate the theory and the limits of its applicability on two datasets.

## 1 Introduction

Sketching is a general probabilistic data compression technique involving random projections (Cormode, 2011). Even routine calculations can be prohibitively computationally expensive if performed on massive datasets. Computational time can be reduced to an acceptable level by allowing some approximation error in the results. Sketching algorithms simplify the computational task by generating a compressed version of the original dataset that then serves as a surrogate for calculations. The compressed dataset is referred to as a sketch, because it acts as a compact representation of the full dataset. Sketching algorithms use a randomized compression stage, which makes them interesting from a statistical viewpoint. Sketching algorithms for linear regression have attracted significant attention in the numerical linear algebra and theoretical computer science communities (Mahoney, 2011; Woodruff, 2014).

To describe sketched regression in more detail, we first assume that the data consist of a length-*n* response vector *y* and an *n* × *p* matrix of covariates, *X*, which is of full rank. It is assumed throughout that *n* > *p*. The objective is to find the least squares coefficients. Given sufficient computational resources, these can be computed exactly as

$$\beta_{\text{F}}={\left(X^{\text{T}}X\right)}^{-1}X^{\text{T}}y,$$

where the subscript F indicates the connection to the full dataset. Only two quantities are needed to determine *β*_F_: the Gram matrix *X* ^T^ *X* and the marginal associations *X* ^T^ *y*. Calculation of *X* ^T^ *X* requires *O*(*np* ^2^) operations, while computation of *X* ^T^ *y* needs only *O* (*np*) calculations. There are two broad methods for sketched regression, namely complete sketching and partial sketching. Complete sketching is based on approximating both *X* ^T^ *X* and *X* ^T^ *y*, whereas partial sketching approximates only the Gram matrix. Drineas et al. (2006) established many important results for complete sketching, and Dhillon et al. (2013) and Pilanci & Wainwright (2016) derived foundational results for partial sketching.

Sketching algorithms use random linear mappings to reduce the size of the dataset from *n* to *k* observations. The random linear mapping can be represented as a *k* × *n* sketching matrix *S*. Complete sketching generates a length-*k* sketched response vector *ỹ* and a *k* × *p* matrix of sketched predictors $\tilde{X}$ . The sketched data are computed through the linear mappings *ỹ* = *Sy* and $\tilde{X}=SX$ . Assuming that $\tilde{X}$ is of rank *p*, the complete sketching estimator *β*_S_ is defined to be the set of least squares coefficients using the sketched responses and predictors,

(1) $$\beta_{\text{S}}={\left({\tilde{X}}^{\text{T}}\tilde{X}\right)}^{-1}{\tilde{X}}^{\text{T}}\tilde{y}.$$

The partial sketching estimator, *β*_P_, is defined as

(2) $$\beta_{\text{P}}={\left({\tilde{X}}^{\text{T}}\tilde{X}\right)}^{-1}X^{\text{T}}y.$$

The key difference between (1) and (2) is that the partial sketching estimator *β*_P_ is constructed using the exact marginal associations *X* ^T^ *y*. Given the sketched data, computation of *β*_S_ or *β*_P_ requires only *O*(*kp* ^2^) operations, compared with the *O*(*np* ^2^) operations required for *β*_F_.

There is a large literature concerned with designing appropriate distributions for the random sketching matrix *S*. Our focus is on data-oblivious random projections, such that the distribution of the sketching matrix is not a function of the source data (*y*, *X*). An example is the Gaussian sketch, where each element is independently distributed as an *N*(0, 1/*k*) variate. We also consider the Hadamard sketch and the Clarkson–Woodruff sketch, random projections that exploit structure and sparsity for computational efficiency.A motivation for this work is that there are no clear ties between data-oblivious random projections and classical subsampling techniques.

Most existing results on the accuracy of sketching are universal worst-case bounds (Woodruff, 2014; Mahoney & Drineas, 2016). This is typical for randomized algorithms; however, a more detailed error analysis can provide important insights (Halko et al., 2011). We investigate the statistical properties of *β*_P_ and *β*_S_ when data-oblivious sketches are used. An important finding is that the signal-to-noise ratio in the source dataset strongly influences the relative efficiency of complete to partial sketching. The statistical analysis also allows the construction of exact confidence intervals for the Gaussian sketch and asymptotic confidence intervals for other random projections, paving the way for their wider use in the statistical community.

At its core, sketched regression is a randomized algorithm for approximate computation of *β*_F_. Repeated application of the sketching algorithm to the same dataset will produce different results. The first stage in our analysis is to establish the distributional properties of the sketching estimators with the source dataset held fixed. An important result is a conditional central limit theorem for the sketched dataset that connects the Hadamard and Clarkson–Woodruff projections to the Gaussian sketch. The conditional analysis of the randomized algorithms is then extended to cover situations where sketching is used for approximate statistical inference. Given a statistical model for the response *y* = *X* *β*_0_ + *ϵ*, with population parameter *β*_0_ and error term *ϵ*, distributional properties of *β*_P_ and *β*_S_ can be determined by integrating over the conditional distributions of the sketching estimators that take *y* and *X* as fixed.

## 2 Background and related work

### 2.1 Preliminaries

We define a number of quantities related to the full dataset before moving on. The total, residual and model sum of squares are given by TSS_F_ = *y* ^T^ *y*, ${\text{RSS}}_{\text{F}}=\|y-X\beta_{\text{F}}{\|}_{2}^{2}$ and ${\text{MSS}}_{\text{F}}=\|X\beta_{\text{F}}{\|}_{2}^{2}$ , respectively, with TSS_F_ = MSS_F_ + RSS_F_. The proportion of variance explained by the model is $R_{\text{F}}^{2}={\text{MSS}}_{\text{F}}/{\text{TSS}}_{\text{F}}$ . These values will be important in characterizing the behaviour of *β*_S_ and *β*_P_. The source data are generically represented by the *n* × *d* matrix *A* = (*y*, *X*).

There are two general categories of distributions for the random matrix *S*: data-aware random projections and data-oblivious random projections. A data-aware random projection uses information in the source data (*y*, *X*) to generate *S*. In contrast, a data-oblivious random projection can be sampled without knowledge of *y* or *X*. Data-aware random projections are closely connected to finite population sampling methods in the statistics literature (Ma & Sun, 2015). Our main focus is on data-oblivious random projections, as their mechanism for data compression is not obviously tied to subsampling. Data-oblivious random projections generate a dataset of *k* pseudo-observations using the source dataset as a component in the generative process.

### 2.2 Data-oblivious sketches

The Gaussian sketch was one of the first projections proposed for sketched regression (Sarlos, 2006). Recall that a Gaussian sketch is formed by independently sampling each element of *S* from an *N*(0, 1/*k*) distribution.A drawback of the Gaussian sketch is that computation of the sketched data is quite demanding, taking *O*(*ndk*) operations. Therefore, work has been done on designing more computationally efficient random projections. Woodruff (2014) gives an excellent survey of work in this area.

The Hadamard sketch is a structured random matrix (Ailon & Chazelle, 2009). The sketching matrix is formed as *S* = Φ *HD* / *√k*, where Φ is a *k* × *n* matrix and *H* and *D* are both *n* × *n* matrices. The fixed matrix *H* is a Hadamard matrix of order *n*. A Hadamard matrix is a square matrix with elements that are either +1 or −1 and orthogonal rows. Although Hadamard matrices do not exist for all integers *n*, the source dataset can be padded with zeros so that a conformable Hadamard matrix is available. The matrix *D* is a diagonal matrix whose *n* diagonal entries are independent Rademacher random variables. The random matrix Φ subsamples *k* rows of *H* with replacement. The structure of the Hadamard sketch allows for fast matrix multiplication, reducing calculation of the sketched dataset to *O*(*nd* log *k*) operations.

The Clarkson–Woodruff sketch is a sparse random matrix (Clarkson & Woodruff, 2013). The projection can be represented as the product of two independent random matrices, *S =* Γ*D*, where Γ is a random *k* × *n* matrix and *D* is a random *n* × *n* matrix. The matrix Γ is initialized as a matrix of zeros. Independently in each column, one element is selected and set to +1. The matrix *D* is a diagonal matrix whose *n* diagonal entries are independent Rademacher random variables. The sparsity of the Clarkson–Woodruff sketch speeds up matrix multiplication, decreasing the complexity of generating the sketched dataset to *O*(*nd*).

## 3 Gaussian sketching

### 3.1 Complete sketching

The Gaussian sketch is mathematically tractable, and it is possible to establish a number of exact finite-sample results regarding the performance of the sketching estimators. In this section we derive the distribution of *β*_S_ in the case where a Gaussian sketch is used. As mentioned previously, all results treat *y* and *X* as fixed. The variability in *β*_S_ is solely due to the use of the random sketching matrix *S*. Let $\left({\tilde{y}}_{j},{\tilde{x}}_{j}^{\text{T}})\;(j=1,\ldots ,k\right)$ refer to the *j*th row of the sketched data matrix $\tilde{A}=(\tilde{y},\tilde{X})$ . Similarly, let $s_{j}^{\text{T}}$ denote the *j*th row of the sketching matrix *S*. The sketched dataset consists of *k* random units $\left({\tilde{y}}_{j},{\tilde{x}}_{j}^{\text{T}})\;(j=1,\ldots ,k\right)$ . The *j*th sketched response is given by ${\tilde{y}}_{j}=s_{j}^{\text{T}}y$ and the *j*th sketched predictor is calculated as ${\tilde{x}}_{j}^{\text{T}}=s_{j}^{\text{T}}X(j=1,\ldots ,k)$ . The *k* sketched instances are independently distributed, because rows of the sketching matrix are independent.

It can be shown that the joint distribution of the sketched data, $p(\tilde{y}\;|\;\tilde{X},y,X)\;p(\tilde{X}|y,X)$ , has the structure of a hierarchical Gaussian linear model. The sketched dataset has a multivariate normal distribution, conditional on the source dataset. This is because the sketched dataset can be expressed as a linear combination of Gaussian random variables. Specifically, row *j* in the sketched dataset is $({\tilde{y}}_{j},{\tilde{x}}_{j}^{\text{T}})=s_{j}^{\text{T}}A$ . Given the source dataset *A* = (*y*,*X*), *A^T^_sj_* is a linear combination of independent Gaussians as *Sj* ~ *N*(0, *I_d_*/*k*), and so $\left({\tilde{y}}_{j},{\tilde{x}}_{j}^{\text{T}}\right)$ must be jointly normally distributed, conditional on the source data *A* = (*y*,*X*). It is easily shownthat the conditional joint distribution of the sketched responses and predictors is then

$$\left(\begin{array}{l} {\tilde{y}}_{j}\\ {\tilde{x}}_{j} \end{array}\right)\;\;|\;Y,\;X\;\sim \;N\;\left\{\;\left(\begin{array}{l} 0\\ 0 \end{array}\right),\;\frac{1}{K}\;\left(\begin{array}{ll} \begin{array}{c} y^{\text{T}}y\\ X^{\text{T}}y \end{array} & \begin{array}{c} y^{\text{T}}X\\ X^{\text{T}}X \end{array} \end{array}\right)\;\right\}\;\;(j=1,\ldots ,k).$$

From standard results on the multivariate normal distribution, it follows that the conditional distribution of *ỹ_j_* given ${\tilde{x}}_{j}$ is also normal with conditional mean $E_{\text{S}}({\tilde{y}}_{j}|{\tilde{x}}_{j},y,X)={\tilde{x}}_{j}^{\text{T}}\beta_{\text{F}}$ . The subscript S is used with the expectation operator to emphasize that the only random quantity is the sketching matrix. The conditional distribution of *ỹ_j_* given the sketched predictors ${\tilde{x}}_{j}$ and the source dataset (*y*, *X*) is

$${\tilde{y}}_{j}|{\tilde{x}}_{j},y,X∼N\left({\tilde{x}}_{j}^{\text{T}}\beta_{\text{F}},\frac{{\text{RSS}}_{\text{F}}}{k}\right)\;(j=1,\ldots ,k).$$

This is the exact form of a standard Gaussian linear model, where the regression coefficient is *β*_F_ and the conditional variance is RSS_F_/*k*. The distribution $p(\tilde{X}|y,X)$ is easily obtained as the marginal distribution of ${\tilde{x}}_{j}$ is also multivariate normal,

$${\tilde{x}}_{j}|y,X∼N\left(0,X^{\text{T}}X/k\right)\;(j=1,\ldots ,k).$$

A Gaussian sketch effectively simulates a series of observations from a Gaussian linear model parameterized in terms of *β*_F_ and RSS_F_, where the design matrix has a matrix normal distribution. The distribution of *β*_S_ conditional on the sketched predictors $\tilde{X}$ follows immediately from standard results on linear models (Searle, 1997, Ch. 3). To obtain the marginal distribution of *β*_S_,it is necessary to integrate over the random sketched design matrix $\tilde{X}$ . Using properties of the normal distribution (Eaton, 2007), it is possible to show that $({\tilde{X}}^{\text{T}}\tilde{X})|y,X∼\text{Wis(}k,X^{\text{T}}X/k\text{)}$ . Hence,

$$({\tilde{X}}^{\text{T}}\tilde{X}{)}^{-1}|y,X∼\text{IW\{}k,k(X^{\text{T}}X{)}^{-1}\text{\},}$$

where iw denotes the inverse Wishart distribution. The marginal distribution of *β*_S_ can then be described using the normal inverse Wishart distribution (Gelman et al., 2014, p. 73). The following theorem characterizes the distribution of *β*_S_ under the Gaussian sketch.

#### Theorem 1

*Suppose β*_S_ *is computed using a Gaussian sketch and that k* ⩾ *p*. Then:

(i) *the conditional distribution of* *β*_S_ *is*
$$\beta_{\text{S}}|\tilde{X},y,X∼N\left\{\beta_{\text{F}},\;\frac{{\text{RSS}}_{\text{F}}}{k}{\left({\tilde{X}}^{\text{T}}\tilde{X}\right)}^{-1}\right\};$$
(ii) *the marginal distribution of* *β*_S_ *is*
$$\beta_{\text{S}}|y,X∼\text{ Student }\left\{\beta_{\text{F}},\frac{{\text{RSS}}_{\text{F}}}{k-p+1}{\left(X^{\text{T}}X\right)}^{-1},\;k-p+1\right\}.$$

For a proof see the Supplementary Material.

An immediate consequence of (i) is the ability to generate exact confidence intervals for the elements of *β*_S_, anapproach that does not seem to have been considered in the existing literature. The variance of *β*_S_,

(3) $$\mathrm{var}\left(\beta_{\text{S}}|y,X\right)=\frac{{\text{RSS}}_{\text{F}}}{\left(k-p-1\right)}(X^{\text{T}}X{)}^{-1},$$

is not dependent on the compression ratio *k*/*n*. Although RSS_F_ can be expected to grow linearly with *n*, this will generally be counterbalanced by (*X* ^T^ *X*)^−1^ decreasing linearly with *n*.

### 3.2 Partial sketching

Partial sketching was first proposed by Dhillon et al. (2013) using uniform subsampling, and was later studied for general sketches by Pilanci & Wainwright (2016). Existing results on partial sketching highlight that the model sum of squares influences the approximation error of the partial sketching estimator *β*_P_. It is easy to see that the variance of the partial sketching estimator will not be a function of the residual sum of squares. From the normal equations it follows that *X* ^T^ *y* = *X* ^T^ *X* *β*_F_. Using this property, we see that conditional on *y* and *X*, the variance of the random linear combination β_P_ = (*X* ^T^ *S* ^T^ *SX*)^−1^ *X* ^T^ *y* = (*X* ^T^ *S* ^T^ *SX*)^−1^ *X* ^T^ *X* *β*_F_ will be a function of the covariates *X* and the fitted values *X* *β*_F_. The residual vector has no influence on the variance of the partial sketching estimator, and as such the variance of *β*_P_ will not be related to the residual sum of squares. This suggests that when the noise level is high, partial sketching may become preferable to complete sketching (Dhillon et al., 2013; Becker et al., 2015).

The hierarchical model for complete sketching provides an intuitive statistical perspective on the mechanics of the algorithm. Partial sketching seems to lack a similar conceptual device. The least squares coefficients can be represented as the solution to the linear system of equations *X* ^T^ *Xb* = *X* ^T^ *y*. Partial sketching simply returns the solution, *b*, to the approximate linear system ${\tilde{X}}^{\text{T}}\tilde{X}b=X^{\text{T}}y$ . Lacking a convenient representation for the estimator, we must proceed in a more pedestrian manner. The mean squared error of the estimator *β*_P_ can be determined using only mean and variance information, and this will be the goal for now. The key observation is that $({\tilde{X}}^{\text{T}}\tilde{X}{)}^{-1}|\;y,X∼\text{IW\{}k,k(X^{\text{T}}X{)}^{-1}\text{\}}$ . Conditional on *y* and *X*, the estimator $\beta_{\text{P}}=({\tilde{X}}^{\text{T}}\tilde{X}{)}^{-1}X^{\text{T}}y$ is a linear combination of the elements of an inverse Wishart random matrix. However, this is a nonstandard distribution, and it is difficult to express directly the distribution function of *β*_P_. Despite this obstacle, it is straightforward to determine the mean and variance of *β*_P_. From properties of the inverse Wishart distribution, it can be seen that the partial sketching estimator is biased, with mean

$$E_{\text{S}}\left(\beta_{\text{P}}|\;y,X\right)=\frac{k}{\left(k-p-1\right)}\beta_{\text{F}},$$

where it is assumed that *k* > *p* + 3. This motivates an alternative unbiased estimator

$$\beta_{\text{P}}^{*}=\frac{\left(k-p-1\right)}{k}{\left({\tilde{X}}^{\text{T}}\tilde{X}\right)}^{-1}X^{\text{T}}y=\frac{\left(k-p-1\right)}{k}\beta_{\text{P}}.$$

Determining the variance of *β*_P_ and the unbiased $\beta_{\text{P}}^{*}$ is a more lengthy computation, which is given in the Supplementary Material. The variance of the unbiased estimator $\beta_{\text{P}}^{*}$ is

(4) $$\mathrm{var}(\beta_{\text{P}}^{*}\;|\;y,X)=\frac{\left(k-p-1\right)}{\left(k-p)\;(k-p-3\right)}\;\left\{{\text{MSS}}_{\text{F}}{\left(X^{\text{T}}X\right)}^{-1}+\frac{k-p+1}{k-p-1}\beta_{\text{F}}\beta_{\text{F}}^{\text{T}}\right\}.$$

By making a connection with method-of-moments estimation it is possible to establish asymptotic normality of both *β*_P_ and $\beta_{\text{P}}^{*}$ as *k* tends to infinity. This motivates the construction of approximate confidence intervals. As the exact variance is unknown, we propose the following estimator of $\mathrm{var}(\beta_{\text{P}}^{*}|y,X)$ using the sketched model sum of squares MSS_S_:

$$\frac{\left(k-p-1\right)}{\left(k-p)\;(k-p-3\right)}\left\{\left(\frac{k-p-1}{k}\right){\text{MSS}}_{\text{S}}{\left({\tilde{X}}^{\text{T}}\tilde{X}\right)}^{-1}+\beta_{\text{P}}^{*}\beta_{\text{P}}^{*\text{T}}\right\}.$$

### 3.3 Relative efficiency

The relative efficiencies of complete and partial sketching are also of interest. As the plug-in estimator *β*_P_ has a greater mean squared error than $\beta_{\text{P}}^{*}$ , it will not be considered in this subsection. The performance of the complete sketching estimator *β*_S_ and the unbiased partial sketching estimator $\beta_{\text{P}}^{*}$ will be compared in terms of mean squared error. As both *β*_S_ and $\beta_{\text{P}}^{*}$ are unbiased, the mean squared errors can be computed using $\mathrm{var}(\beta_{\text{S}}|y,X)$ and $\mathrm{var}(\beta_{\text{P}}^{*}|y,X)$ . Comparing (3) and (4), it can be seen that the variance of $\beta_{\text{P}}^{*}$ is dependent on MSS_F_ whereas the variance of *β*_S_ is dependent on RSS_F_. This suggests that the signal-to-noise ratio in the source dataset will be an influential factor in determining which estimator is more efficient. In the Supplementary Material it is shown that for *k* > *p* + 3 the relative efficiency can be bounded in terms of the signal-to-noise ratio

$$\frac{R_{\text{F}}^{2}}{1-R_{\text{F}}^{2}}\;⩽\;\frac{E_{\text{S}}(\|\beta_{\text{P}}^{*}-\beta_{\text{F}}{\|}_{2}^{2}|y,X)}{E_{\text{S}}(||\beta_{\text{S}}-\beta_{\text{F}}{\|}_{2}^{2}y,X)}\;⩽\;\frac{2(k-p-1)}{\left(k-p-3\right)}\frac{R_{\text{F}}^{2}}{1-R_{\text{F}}^{2}}.$$

When $R_{\text{F}}^{2}$ is close to 1, complete sketching can be orders of magnitude more efficient than partial sketching; and when $R_{\text{F}}^{2}$ is close to 0, partial sketching can be orders of magnitude more efficient than complete sketching.

### 3.4 Combined estimator

So far we have assumed that an analyst must choose between one of the two methods; but obtaining both $\beta_{\text{P}}^{*}$ and *β*_S_ from a single sketch is computationally cheap and may be an attractive strategy. The most demanding operation with the sketched data is calculating $({\tilde{X}}^{\text{T}}\tilde{X}{)}^{-1}$ . Given this quantity, it is economical to compute both *β*_S_ and $\beta_{\text{P}}^{*}$ . Becker et al. (2015) mentioned that they were investigating such a strategy, but did not give any details. Our development of a combined estimator is motivated by the fact that, even when using a single sketch $\left(\tilde{y},\tilde{X}\right)$ , the two estimators are uncorrelated, i.e., $\mathrm{cov}(\beta_{\text{P}}^{*},\beta_{\text{S}}\;|\;y,X)=0$ . This is established in the Supplementary Material by taking iterated expectations and using the hierarchical model from § 3.1. Asimple strategy is then to take a weighted combination of *β*_S_ and $\beta_{\text{P}}^{*}$ . A combined estimator *β*C can be defined as

$$\beta_{\text{C}}=\phi \beta_{\text{S}}+(1-\phi )\beta_{\text{P}}^{*}$$

for some 0 ⩽ *φ* ⩽ 1. The value of *φ* that minimizes the mean squared error is $\phi_{\text{opt}}=\text{tr}\{\mathrm{var}(\beta_{\text{P}}^{*})\;|\;y,X\}/[\text{tr}\{\mathrm{var}(\beta_{\text{P}}^{*}\;|\;y,X)\}+\text{tr\{}\mathrm{var}(\beta_{\text{S}}|y,X)\text{\}}]$ . Use of the weighted estimator is expected to be most beneficial when the signal-to-noise ratio is moderate, i.e., $R_{\text{F}}^{2}\approx 0.5$ . When the signal-to-noise ratio is either very high or very low, there is little advantage in using the weighted estimator, as either the complete or the partial estimator will dominate.

### 3.5 One-step correction

As noted by a referee, the combined estimator is related to another strategy in the sketching literature for improving *β*_S_. Dhillon et al. (2013) and Pilanci & Wainwright (2016) proposed a refinement procedure that uses gradient information from the source dataset. The one-step corrected estimator is defined as

(5) $$\beta_{\text{H}}=\beta_{\text{S}}+({\tilde{X}}^{\text{T}}\tilde{X}{)}^{-1}X^{\text{T}}(y-X\beta_{\text{S}})=\left\{I-{\left({\tilde{X}}^{\text{T}}\tilde{X}\right)}^{-1}X^{\text{T}}X\right\}\beta_{\text{S}}+({\tilde{X}}^{\text{T}}\tilde{X}{)}^{-1}X^{\text{T}}y.$$

Now the least squares solution *β*_F_ satisfies *X* ^T^(*y* – *X* *β*_F_) = 0, so

(6) $$\beta_{\text{F}}=\beta_{\text{F}}+({\tilde{X}}^{\text{T}}\tilde{X}{)}^{-1}X^{\text{T}}(y-X\beta_{\text{F}})=\left\{I-{\left({\tilde{X}}^{\text{T}}\tilde{X}\right)}^{-1}X^{\text{T}}X\right\}\beta_{\text{F}}+({\tilde{X}}^{\text{T}}\tilde{X}{)}^{-1}X^{\text{T}}y.$$

Subtracting (6) from (5) gives the following expression for the error:

(7) $$\beta_{\text{H}}-\beta_{\text{F}}=\{I-({\tilde{X}}^{\text{T}}\tilde{X}{)}^{-1}X^{\text{T}}X\}(\beta_{\text{S}}-\beta_{\text{F}}).$$

The one-step estimator can be interpreted as a single step of the iterative Hessian sketch proposed by Pilanci & Wainwright (2016), initialized at *β_S_*. Setting $\tilde{H}=({\tilde{X}}^{\text{T}}\tilde{X}{)}^{-1}X^{\text{T}}X$ it follows from (7) and Theorem 1(i) that

(8) $$E_{\text{S}}(\|\beta_{\text{H}}-\beta_{\text{F}}{\|}_{2}^{2}|y,X)=E_{\tilde{X}}\left[\text{tr\{}k^{-1}{\text{RSS}}_{\text{F}}{\left({\tilde{X}}^{\text{T}}\tilde{X}\right)}^{-1}{\left(I-\tilde{H}\right)}^{\text{T}}(I-\tilde{H})\text{\}}\right].$$

The key terms in (8) are the random matrices $({\tilde{X}}^{\text{T}}\tilde{X}{)}^{-1}$ and $\tilde{H}=({\tilde{X}}^{\text{T}}\tilde{X}{)}^{-1}X^{\text{T}}X$ . As $({\tilde{X}}^{\text{T}}\tilde{X}{)}^{-1}\;|\;y,X∼\text{IW\{}k,k(X^{\text{T}}X{)}^{-1}\text{\}}$ , it is possible to evaluate the expectation in (8) using the first, second and third moments of the inverse Wishart distribution. The exact expression for (8) is lengthy and is given in the Supplementary Material. The main conclusions are that the one-step estimator *β*_H_ can have a larger mean squared error than *β*_S_ when the ratio *k*/*p* of sketch size to number of variables is close to 1. As *k*/*p* increases, the one-step estimator becomes more efficient than both *β*_S_ and *β*_C_ with the optimal weight *φ*_opt_. The relative efficiency of *β*_C_ to *β*_S_ is at most 2. The relative efficiency of *β*_H_ to *β*_S_ can be much higher, provided that *k*/*p* is sufficiently large.

## 4 Asymptotics

### 4.1 Preliminaries

Finite-sample distributions of random projection estimators can be mathematically intractable, and thus asymptotic analysis can be a powerful tool (Diaconis & Freedman, 1984; Li et al., 2006). It is very difficult to establish meaningful finite-sample results for the Hadamard and Clarkson– Woodruff sketches, as they are discrete distributions over an enormous combinatorial space. Instead, it is useful to study the large-*n* distribution of the estimators *β*_S_ and *β*_P_ to obtain an interpretable expression.

As *β*_F_ is the estimand in sketching algorithms, conditioning on the source data is required in the asymptotic analysis. To elaborate, let *A*_(*n*)_ = (*y*_(*n*)_, *X*_(*n*)_) represent the *n*×*d* source data matrix of full column rank.Any source data matrix *A*_(*n*)_ has a set of associated least squares coefficients, which will be denoted by $\beta_{\text{F}}^{\left(n\right)}$ here. The overall goal is to determine the asymptotic form of the distributions $p(\beta_{\text{S}}|A_{\left(n\right)})$ and $p(\beta_{\text{P}}^{*}|A_{\left(n\right)})$ for some arbitrary large dataset *A*(*n*). To take limits, we employ a fixed sequence of *n* × *d* datasets, all of rank *d*.

Some related work has been done by Ma et al. (2015), who developed Taylor series approximations for the bias and variance of data-aware sketched regression estimators, where the asymptotic expansion is taken in the sketch size *k*. In independent work, Dobriban & Liu (2019) examined the behaviour of data-oblivious sketching algorithms in the asymptotic regime where *k*, *d* →∞, using elements of random matrix theory. Our work is novel, as we study data-oblivious random projections in the regime where *k* and *d* are fixed, while taking limits in the number *n* of source observations.

### 4.2 Sketching central limit theorem

A central limit theorem for sparse sketching matrices with independent entries is given in Li et al. (2006). The Clarkson–Woodruff sketch and the Hadamard sketch have dependent entries, so we use a different method of proof. Under some regularity conditions, the Hadamard and Clarkson–Woodruff sketches produce sketched data that asymptotically have the same matrix normal distribution as under the Gaussian sketch.

The *k × d* random matrix *Ã* is the output of a stochastic process governed by the fixed *n* × *d* source dataset *A*_(*n*)_ and the distribution of the random *k* × *n* sketching matrix *S*. Eachcolumnof the sketched dataset is a linear combination of random vectors, the number of which increases with *n*. Under an assumption on the limiting leverage scores of the source data matrix, we can establish a central limit theorem for the sketched dataset. The leverage scores of the observations in the source data matrix have been identified as an important structural property of sketching algorithms (Mahoney & Drineas, 2016). Assumption 1 highlights their role in establishing asymptotic normality of the sketched data matrix.

#### Assumption 1

Let $A_{\left(n\right)}=U_{\left(n\right)}D_{\left(n\right)}V_{\left(n\right)}^{\text{T}}$ be the singular value decomposition of the *n* × *d* source dataset, and let $u_{(n)i}^{\text{T}}$ be the *i*th row in *U*(*n*). The maximum leverage score tends to zero, that is,

$$\underset{n\to \infty }{\lim}\;\underset{i=1,\ldots ,n}{\max}\|u_{(n)i}{\|}_{2}^{2}=0.$$

Theorem 2 is the sketching central limit theorem. Its proof is given in the Supplementary Material.

#### Theorem 2

*Consider a sequence of arbitrary n* × *d data matrices A*(*n*), *where d is fixed*. *Let $A_{\left(n\right)}=U_{\left(n\right)}D_{\left(n\right)}V_{\left(n\right)}^{\text{T}}$ be the singular value decomposition of A*(*n*), *and let S be a k* × *n Hadamard or Clarkson–Woodruff sketching matrix where k is also fixed. Suppose that Assumption 1 is satisfied. Then, as n tends to infinity, the following convergence in distribution holds*:

$$\{\tilde{A}V_{\left(n\right)}D_{\left(n\right)}^{-1}|A_{\left(n\right)}\}\to \text{MN}\left(0,I_{k},I_{d}/k\right),$$

*where* MN *denotes the matrix normal distribution*.

### 4.3 Sketching estimators

The central limit theorem for the sketched data suggests that the results on *β*_S_ and *β*_P_ for the Gaussian sketch will also hold approximately for the Hadamard and Clarkson–Woodruff sketches for large *n*. To establish convergence of the estimators it helps to make an extra assumption on the sequence of source datasets.

#### Assumption 2

We have that

$$\underset{n\to \infty }{\lim}\;n^{-1}\left(\begin{array}{ll} \begin{array}{c} y_{\left(n\right)}^{\text{T}}y_{\left(n\right)}\\ X_{\left(n\right)}^{\text{T}}y_{\left(n\right)} \end{array} & \begin{array}{c} y_{\left(n\right)}^{\text{T}}X_{\left(n\right)}\\ X_{\left(n\right)}^{\text{T}}X_{\left(n\right)} \end{array} \end{array}\right)=\;Q$$

for some positive-definite matrix *Q*.

The limiting matrix *Q* allows one to avoid specifying a probability model for the source dataset, without overcomplicating the mathematical analysis. Under Assumptions 1 and 2, it is possible to establish an asymptotic result for *β*_S_ and *β*_P_.

#### Theorem 3

*Suppose that Assumptions* 1 *and* 2 *hold, k ⩾ p, and β*_S_ *is computed using a Hadamard or Clarkson–Woodruff sketch*. Let $({\tilde{X}}^{\text{T}}\tilde{X}{)}^{+}$ *denote the Moore–Penrose pseudo-inverse of* $\left({\tilde{X}}^{\text{T}}\tilde{X}\right)$ . *Let*

$${\tilde{C}}_{\left(n\right)}=\frac{{\text{RSS}}_{\text{F}}^{\left(n\right)}}{k}({\tilde{X}}^{\text{T}}\tilde{X}{)}^{+},\;C_{\left(n\right)}=\frac{{\text{RSS}}_{\text{F}}^{\left(n\right)}}{k-p+1}(X_{\left(n\right)}^{\text{T}}X_{\left(n\right)}{)}^{-1}\;.$$

*Then, as n* → ∞, *the following convergence results hold in distribution*:

(i)
$\left\{C_{\left(n\right)}^{-1/2}\;(\beta_{\text{S}}-\beta_{\text{F}}^{\left(n\right)})\;|A_{\left(n\right)}\}\to \text{Student}(0,I_{p},k-p+1\right)$

(ii)
$\left\{{\tilde{C}}_{\left(n\right)}^{-1/2}(\beta_{\text{S}}-\beta_{\text{F}}^{\left(n\right)})\;|A_{\left(n\right)}\}\to N(0,I_{p}\right)$

The proof is given in the Supplementary Material. For large *n* we expect *β*_S_ to be approximately distributed as per Theorem 1 for both the Hadamard and the Clarkson–Woodruff sketches.

It is harder to establish a comparable limit theorem for $\beta_{\text{P}}^{*}$ , because of the nonstandard distribution of $\beta_{\text{P}}^{*}$ when a Gaussian sketch is used. Instead, we wish to show that the partial sketching estimators under the Hadamard and Clarkson–Woodruff sketches have similar mean and variance properties to the Gaussian partial sketching estimator. Convergence in moments can be established given a stability condition on the singular values of the sketched data matrix.

#### Assumption 3

The sequence of source datasets is such that $E_{\text{S}}\{1/\sigma_{\min}^{4}(n^{-1}{\tilde{X}}^{\text{T}}\tilde{X})|y,X\}$ is finite for large enough *n*, where σ_min_(·) denotes the minimum singular value of a matrix.

This additional regularity condition enables a formal limit theorem regarding the moments of $\beta_{\text{P}}^{*}$ to be established.

#### Theorem 4

*Suppose that Assumptions* 1*–*3 *hold, k* > *p* + 3, *and* $\beta_{\text{P}}^{*}$ *is computed using a Hadamard or Clarkson–Woodruff sketch*. *Let*

$$C_{\left(n\right)}=\frac{\left(k-p-1\right)}{\left(k-p)(k-p-3\right)}\;\left\{{\text{MSS}}_{\text{F}}^{\left(n\right)}{\left(X_{\left(n\right)}^{\text{T}}X_{\left(n\right)}\right)}^{-1}+\frac{k-p+1}{k-p-1}\beta_{\text{F}}^{\left(n\right)}\beta_{\text{F}}^{(n)\text{T}}\right\}.$$

*Then, as n* → ∞:

(i)
$E_{\text{S}}\{\beta_{\text{P}}^{*}-\beta_{\text{F}}^{\left(n\right)}|A_{\left(n\right)}\}\to 0$

(ii)
${\mathrm{var}}_{\text{S}}\{C_{\left(n\right)}^{-1/2}(\beta_{\text{P}}^{*}-\beta_{\text{F}}^{\left(n\right)})|A_{\left(n\right)}\}\to I_{p}$

The proof is given in the Supplementary Material. This theorem suggests that the conditional bias and variance of $\beta_{\text{P}}^{*}$ under the Clarkson–Woodruff and Hadamard sketches should be approximately equal to those under the Gaussian sketch. The results here are meant to provide useful heuristics for assessing the uncertainty associated with the output of the randomized approximation algorithm. There is a need to quantify the approximation error of sketching algorithms and communicate it to end users (Lopes et al., 2018), for which the asymptotic results developed in this section may be helpful.

## 5 Unconditional results

The previous analysis treated the source dataset as fixed to isolate the approximation error introduced by the random projection. When sketching is used for statistical inference, the hierarchical model of § 3.1 can be extended to include a source of variation at the population level. We take the design matrix *X* to be fixed and treat the response *y* as random. The assumed data-generating process is *y* = *X* *β*_0_ + *ε*, where *ε* is a vector of *n* independent and identically distributed random variables with mean zero and variance *σ* ^2^. Let *γ* ^2^ represent the average mean function sum of squares, so $\gamma^{2}=\;||X\beta_{0}{\|}_{2}^{2}/n$ . As shown in Searle (1997), at the population level the ordinary least squares estimator satisfies $E_{y}\left(\beta_{\text{F}}|X\right)=\beta_{0},{\mathrm{var}}_{y}\left(\beta_{\text{F}}|X\right)=\sigma^{2}{\left(X^{\text{T}}X\right)}^{-1}E_{y}\left({\text{RSS}}_{\text{F}}|X\right)=(n-p)\sigma^{2}$ and $E_{y}\left({\text{MSS}}_{\text{F}}|X\right)=p\sigma^{2}+n\gamma^{2}$ . Taking iterated expectations, it can be seen that the Gaussian sketch gives an unbiased estimator of the population parameter $\beta_{0}:E_{y}\left(\beta_{\text{S}}\;|\;X\right)=E_{y}\left\{E_{\text{S}}\left(\beta_{\text{S}}\;|\;y,X\right)\right\}=E_{y}\left(\beta_{\text{F}}\;|\;X\right)=\beta_{0}$ . The same argument shows that $E_{y}\left(\beta_{\text{p}}^{*}\;|\;X\right)=\beta_{0}$ . In the Supplementary Material, we use the law of total variance to determine the unconditional variances

$$\begin{array}{lll} {\mathrm{var}}_{y}(\beta_{\text{S}}\;|\;X) & = & \sigma^{2}{\left(X^{\text{T}}X\right)}^{-1}+\frac{(n-p)\sigma^{2}}{\left(k-p-1\right)}{\left(X^{\text{T}}X\right)}^{-1},\\ {\mathrm{var}}_{y}(\beta_{\text{P}}^{*}\;|\;X) & = & \sigma^{2}{\left(X^{\text{T}}X\right)}^{-1}+\;\frac{\left(k-p-1\right)}{\left(k-p)(k-p-3\right)}\;[(p\sigma^{2}+n\gamma^{2})\;{\left(X^{\text{T}}X\right)}^{-1}+\;\frac{k-p+1}{k-p-1}\{\sigma^{2}{\left(X^{\text{T}}X\right)}^{-1}+\beta_{0}\beta_{0}^{\text{T}}\}]. \end{array}$$

For large *n*, the most significant term in the unconditional variance of *β*_S_ is *n*σ^2^(*X* ^T^ *X*)^−1^. The dominating term in the unconditional variance of $\beta_{\text{P}}^{*}$ is *nγ* ^2^(*X* ^T^ *X*)^−1^, a function of the average model sum of squares γ ^2^. We reach conclusions similar to those of the conditional analysis in § 3.3, in that *β*_S_ is expected to be more efficient when the signal-to-noise ratio is high, while $\beta_{\text{P}}^{*}$ is expected to be more efficient when the signal-to-noise ratio is low. Under Assumptions 1–3, the variance expressions give asymptotic approximations for the Hadamard and Clarkson–Woodruff projections. These results can be extended to account for more complicated error models on *ϵ* if it is still possible to determine $E_{y}\left(\beta_{\text{F}}\;|\;X\right),{\mathrm{var}}_{y}\left(\beta_{\text{F}}\;|X\right),E_{y}\left({\text{RSS}}_{\text{F}}\;|\;X\right)\;$ and $E_{y}\left({\text{MSS}}_{\text{F}}\;|\;X\right)$ . Raskutti & Mahoney (2016) provides further results on the performance of sketching estimators from an inferential perspective.

## 6 Data application

### 6.1 Human leukocyte antigen locus dataset

We compare the performance of the sketching estimators on a genetic dataset from the UK Biobank database.We use a small extract of the data in Astle et al. (2016). The selected response variable is mean red cell volume, taken from the full blood count assay and with adjustments for various technical and environmental covariates. Genome-wide imputed genotype data in expected allele dose format were available on *n* = 132 353 study subjects (Howie et al., 2009; Bycroft etal., 2018). We consider 1000 genetic variants in the human leukocyte antigen, HLA, region of chromosome 6, selected so that no pair of variants had squared Pearson correlation of posterior expected allele doses greater than 0.8. We chose to focus on this region because many associations have been discovered in a genome-wide scan using univariable models; these associations were with variants having different allele frequencies, which suggests multiple distinct causal variants in the region. The aim is to perform a multivariable regression analysis to obtain variant effect size estimates that are conditional on the other variants in the region.

**Fig. 1:**
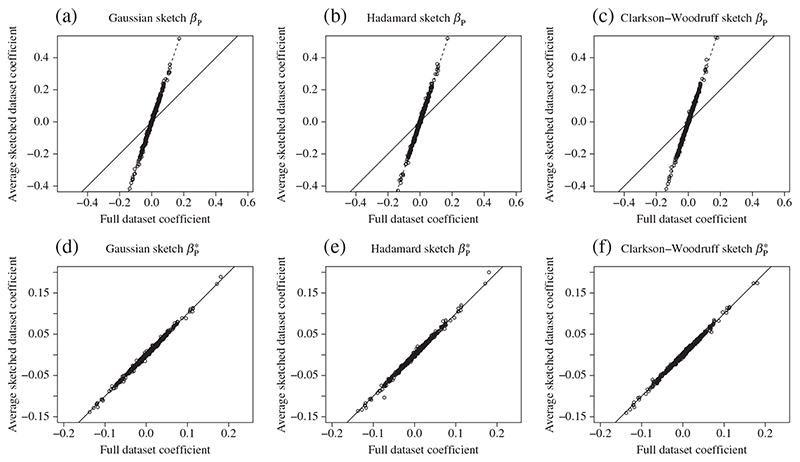
Bias of partial sketching estimators on the HLA dataset: panels (a)–(c) show results for *β*_P_ and panels (d)– (f) results for the bias-corrected estimator $\beta_{\text{P}}^{*}$ ; mean estimates are plotted against the true values. In this scenario *n* = 132 353, *p* = 1000 and *k* = 1500. The solid line in each panel is the identity line, and the dashed line in panels (a)–(c) represents the theoretical bias factor.

An early theoretical finding was that the partial sketching estimator *β*_P_ is biased. One thousand sketches were taken to estimate the bias $E_{\text{S}}\left(\beta_{\text{P}}-\beta_{\text{F}}|y,X\right)$ with *k* = 1500. We also computed the bias-corrected estimator $\beta_{\text{P}}^{*}$ in each replication. Figure 1 plots the average value of the estimators against the true value of the least squares coefficient using the full dataset. The top row shows results for *β*_P_, and the bottom row shows results for $\beta_{\text{P}}^{*}$ . The left, middle and right columns display results for the Gaussian, Hadamard and Clarkson–Woodruff sketches, respectively. The solid line in each panel is the identity line. The dashed line in the top row represents the theoretical bias, with slope *k*/(*k* – *p* – 1).

The results in panels (a)–(c) show that *β*_P_ is biased for each of the random projections. The bias closely matches the theoretical factor. Panels (d)–(f) show that the adjusted estimator $\beta_{\text{P}}^{*}$ appears to be unbiased, with the mean values falling close to the identity line.

We also compared the complete and partial sketching estimators in terms of mean squared error and coverage of confidence intervals at *k* = 1500 and *k* = 10 000. Moreover, we compared the data-oblivious sketches to simple uniform subsampling with replacement. Table 1 reports the mean squared error for each of the estimators. The signal-to-noise ratio is quite low for this dataset, with $R_{\text{F}}^{2}=0.02$ . We expect that partial sketching will be much more efficient than complete sketching on this dataset given the low signal-to-noise ratio. The simulation results support this prediction, with $\beta_{\text{P}}^{*}$ having a mean squared error roughly 60 times smaller than *β*_S_ at both values of *k*. The results are very similar for each of the random projections, suggesting that the asymptotic approximations are reasonable for this dataset. For *k* = 1500, the mean squared error of *β*_P_ is approximately 10 times that of $\beta_{\text{P}}^{*}$ .For *k* = 10 000 there is less of a difference, as the ratio *k*/(*k*−*p*−1) is closer to 1.

Table 2 summarizes the coverage of 95% confidence intervals forthe sketching estimators. We report the overall proportion of intervals containing the true value of the least squares estimate *β*_F_ over the 250 sketches and *p* = 1000 coefficients. The observed coverage is close to the nominal level of 0.95 at both levels of *k*. The different random projections give very similar results, suggesting that the use of asymptotic approximations is again reasonable for this dataset. The intervals for the Hadamard sketch appear to be slightly conservative at *k* = 10 000.

Table 3 reports the average sketching times for the data-oblivious sketches. We computed 10 sketches using each projection. The Gaussian sketch is an order of magnitude slower than the Hadamard projection and two orders of magnitude slower than the Clarkson–Woodruff sketch.

### 6.2 New York flights dataset

We also evaluated the sketching algorithms on the New York flights dataset available in the R (R Development Core Team, 2021) package nycflights13 (Wickham, 2014). Arrival delay was taken as the response, and departure delay, distance, departure time, origin, and month and day were chosen to be the covariates. Rows of the dataset with missing data were omitted, sothat we were left with *n* = 327 346 and *d* = 47. The goal is to compare the accuracy of the various sketches on real data rather than to build a statistical model for the flights dataset. We compare the mean squared error of the estimators and the coverage of confidence intervals for *k* = 5000. In contrast to the HLA dataset, the flights dataset has a very high $R_{\text{F}}^{2}$ value of 0.99. We took 500 sketches to compare complete and partial sketching. See Table 4 for details.

## 7 Discussion

In recent years work has been done to adapt sketching methods for statistical inference in large datasets, building upon the worst-case bounds developed in the computer science literature. Geppert et al. (2017) and Bardenet & Maillard (2015) investigated sketching algorithms for Bayesian regression, and derived bounds on the difference between the sketched posterior distribution and the full-data posterior distribution. Only complete sketching was considered in those works. The results on the advantages of partial sketching in this paper could motivate adaptations that make use of the exact marginal associations *X* ^T^ *y*. Sketching ideas have been used to develop methods for approximate nonlinear regression (Banerjee et al., 2013; Avron et al., 2014). The goodness of fit ofthe model may also influence the relative efficiency of different sketching algorithms in more complex regression tasks. A related branch of work uses random projections to reduce the number of predictors inregressionandclassificationproblems(Shah & Meinshausen, 2018; Guhaniyogi & Dunson, 2015; Cannings & Samworth, 2017).

## Supplementary Material

Supplementary material available at Biometrika online includes proofs of all the theorems.

Supplementary Material

## Figures and Tables

**Table 1:** Mean squared errors of sketching estimators on the HLA dataset

	k = 1500			k = 10 000		
	*β*_S_	*β*_P_	$\beta_{\text{P}}^{*}$	*β*_S_	*β*_P_	$\beta_{\text{P}}^{*}$
Gaussian	238 (3)	39 (0.7)	3.8 (0.08)	13.3 (0.17)	0.28 (0.004)	0.21 (0.002)
Hadamard	238 (4)	39 (0.7)	3.8 (0.07)	12.5 (0.16)	0.26 (0.003)	0.20 (0.002)
Clarkson–Woodruff	241 (3)	38 (0.8)	4.0 (0.05)	13.2 (0.16)	0.28 (0.004)	0.21 (0.002)
Uniform	375 (15)	105 (7.6)	10.7 (0.55)	13.8 (0.20)	0.38 (0.007)	0.29 (0.005)

**Table 2:** Coverage of confidence intervals; the largest standard error is 0.004

	HLA		HLA		Flights	
	*k* = 1500		*k* = 10 000		*k* = 1500	
	*β*_S_	$\beta_{\text{P}}^{*}$	*β*_S_	$\beta_{\text{P}}^{*}$	*β*_S_	$\beta_{\text{P}}^{*}$
Gaussian	0.950	0.953	0.950	0.951	0.948	0.951
Hadamard	0.949	0.949	0.954	0.954	0.950	0.948
Clarkson–Woodruff	0.947	0.952	0.951	0.950	0.948	0.947

**Table 3:** Timings for sketching: average times to compute the sketched dataset *Ã* = *SA*, in seconds

	HLA		Flights
	*k* = 1500	*k* = 10 000	*k* = 5000
Gaussian	522	3479	404
Hadamard	57	65	5.8
Clarkson–Woodruff	5.3	5.4	0.2

**Table 4:** Mean squared errors of sketching estimators (with standard errors in parentheses) on the flights dataset with *k* = 5000

	*β*_S_	*β*_P_	$\beta_{\text{P}}^{*}$
Gaussian	60 (2)	14900 (400)	14900 (400)
Hadamard	63 (2)	14800 (500)	13900 (400)
Clarkson–Woodruff	66 (2)	15000 (500)	13800 (400)
Uniform	64 (2)	14600 (500)	14600 (400)

## References

[R1] Ailon N, Chazelle B (2009). The fast Johnson Lindenstrauss transform and approximate nearest neighbors. SIAM J Comp.

[R2] Astle WJ, Elding H, Jiang T, Allen D, Ruklisa D, Mann AL, Mead D, Bouman H, Riveros-Mckay F, Kostadima MA (2016). The allelic landscape of human blood cell trait variation and links to common complex disease. Cell.

[R3] Avron H, Nguyen H, Woodruff D (2014). Subspace embeddings for the polynomial kernel.

[R4] Banerjee A, Dunson DB, Tokdar ST (2013). Efficient Gaussian process regression for large datasets. Biometrika.

[R5] Bardenet R, Maillard O-A (2015). A note on replacing uniform subsampling by random projections in MCMC for linear regression of tall datasets.

[R6] Becker S, Kawas B, Petrik M, Ramamurthy K (2015). Robust partially-compressed least-squares. arXiv.

[R7] Bycroft C, Freeman C, Petkova D, Band G, Elliott LT, Sharp K, Motyer A, Vukcevic D, Delaneau O, O’Connell J (2018). The UK Biobank resource with deep phenotyping and genomic data. Nature.

[R8] Cannings TI, Samworth RJ (2017). Random-projection ensemble classification. J R Statist Soc B.

[R9] Clarkson KL, Woodruff DP (2013). Low rank approximation and regression in input sparsity time.

[R10] Cormode G (2011). Foundations and Trends in Databases.

[R11] Dhillon P, Lu Y, Foster DP, Ungar L (2013). New subsampling algorithms for fast least squares regression.

[R12] Diaconis P, Freedman D (1984). Asymptotics of graphical projection pursuit. Ann Statist.

[R13] Dobriban E, Liu S (2019). Asymptotics for sketching in least squares regression.

[R14] Drineas P, Mahoney MW, Muthukrishnan S (2006). Sampling algorithms for ℓ_2_ regression and applications.

[R15] Eaton ML (2007). Multivariate Statistics: A Vector Space Approach.

[R16] Gelman A, Carlin JB, Stern HS, Dunson DB, Vehtari A, Rubin DB (2014). Bayesian Data Analysis.

[R17] Geppert LN, Ickstadt K, Munteanu A, Quedenfeld J, Sohler C (2017). Random projections for Bayesian regression. Statist Comp.

[R18] Guhaniyogi R, Dunson DB (2015). Bayesian compressed regression. J Am Statist Assoc.

[R19] Halko N, Martinsson PG, Tropp JA (2011). Finding structure with randomness: Probabilistic algorithms for constructing approximate matrix decompositions. SIAM Rev.

[R20] Howie BN, Donnelly P, Marchini J (2009). A flexible and accurate genotype imputation method for the next generation of genome-wide association studies. PLoS Genet.

[R21] Li P, Hastie TJ, Church KW (2006). Very sparse random projections.

[R22] Lopes M, Wang S, Mahoney M, Dy J, Krause A (2018). Error estimation for randomized least-squares algorithms via the bootstrap.

[R23] Ma P, Mahoney MW, Yu B (2015). A statistical perspective on algorithmic leveraging. J Mach Learn Res.

[R24] Ma P, Sun X (2015). Leveraging for big data regression. WIREs Comp Statist.

[R25] Mahoney M (2011). Foundations and Trends in Machine Learning.

[R26] Mahoney M, Drineas P, Buhlmann P, Drineas P, Kane M, van de Laan M (2016). Handbook of Big Data.

[R27] Pilanci M, Wainwright MJ (2016). Iterative Hessian sketch: Fast and accurate solution approximation for constrained least-squares. J Mach Learn Res.

[R28] R Development Core Team (2021). R: A Language and Environment for Statistical Computing.

[R29] Raskutti G, Mahoney MW (2016). A statistical perspective on randomized sketching for ordinary least-squares. J Mach Learn Res.

[R30] Sarlos T (2006). Improved approximation algorithms for large matrices via random projections.

[R31] Searle SR (1997). Linear Models.

[R32] Shah RD, Meinshausen N (2018). Min-wise hashing for large-scale regression and classification with sparse data. arXiv.

[R33] Wickham H (2014). nycflights13: Flights that Departed NYC in 2013.

[R34] Woodruff DP (2014). Foundations and Trends in Theoretical Computer Science.

