# Supplementary material for: Statistical properties of sketching algorithms
Source: Biometrika. Author manuscript; Available in PMC 2022 Feb 4. (PMC7612324; doi:10.1093/biomet/asaa062)
Supplement: Supplementary Material [file EMS140909-supplement-Supplementary_Material.zip › supplementary_v3.pdf]

Biometrika (2017), **103**, 1, *pp.* 1–23
Printed in Great Britain Advance Access publication on 31 July 2016

Supplementary material for 'Statistical properties of sketching algorithms'

BY D. C. AHFOCK, W. J. ASTLE AND S. RICHARDSON
MRC Biostatistics Unit,
MRC Biostatistics Unit, University of Cambridge
d.ahfock@uq.edu.au

1. PROOF OF THEOREM 1

We use the following lemma about the Normal Inverse-Wishart distribution in many of our results (Gelman et al., 2014, p.73).

LEMMA S.1. *Suppose that Σ is a random $d \times d$ matrix and y is a d-dimensional random vector from the following hierarchical model*

$$y \mid \Sigma \sim N(\mu, \Sigma/\kappa), \qquad \Sigma \sim \text{Inverse-Wishart}(\Lambda, \nu),$$

where Λ is a $d \times d$ scale matrix, ν is a scalar giving degrees of freedom, and κ is a scaling constant. Then marginally,

$$y \sim \text{Student}(\mu,\ \Lambda/\{\kappa(\nu - d + 1)\},\ \nu - d + 1).$$

Theorem 1 (ii) follows from setting $\mu = \beta_F$, $\Sigma = (\widetilde{X}^{\mathrm{T}}\widetilde{X})^{-1}$, $\kappa = k/RSS_F$, $\Lambda = k(X^{\mathrm{T}}X)^{-1}$, $\nu = k$ and $d = p$. Theorem 1 (i) follows from standard results on linear models, for example see Searle (1997, Chapter 3).

2. VARIANCE FOR PARTIAL SKETCHING

Let the singular value decomposition of X be given by $X = UDV^{\mathrm{T}}$. The singular value decomposition will help to simplify expressions in later working. The sketched Gram matrix has the form $\widetilde{X}^{\mathrm{T}}\widetilde{X} = VDU^{\mathrm{T}}S^{\mathrm{T}}SUDV^{\mathrm{T}}$. As $U^{\mathrm{T}}S^{\mathrm{T}}SU \sim \text{Wishart}(k, I_p/k)$, the matrix $U^{\mathrm{T}}S^{\mathrm{T}}SU$ is almost surely invertible provided $k \geq p$. The inverse Gram matrix can then be written as

$$(\widetilde{X}^{\mathrm{T}}\widetilde{X})^{-1} = (DV^{\mathrm{T}})^{-1}(U^{\mathrm{T}}S^{\mathrm{T}}SU)^{-1}(VD)^{-1} = VD^{-1}(U^{\mathrm{T}}S^{\mathrm{T}}SU)^{-1}D^{-1}V^{\mathrm{T}}.$$

The expression for β_P can then be simplified to

$$\beta_P = VD^{-1}(U^{\mathrm{T}}S^{\mathrm{T}}SU)^{-1}D^{-1}V^{\mathrm{T}}X^{\mathrm{T}}y = VD^{-1}(U^{\mathrm{T}}S^{\mathrm{T}}SU)^{-1}D^{-1}V^{\mathrm{T}}VDU^{\mathrm{T}}y = VD^{-1}(U^{\mathrm{T}}S^{\mathrm{T}}SU)^{-1}U^{\mathrm{T}}y.$$

© 2018 Biometrika Trust

Let $M = (U^{\mathrm{T}}S^{\mathrm{T}}SU)^{-1}$. We know that $M \sim$ Inverse-Wishart(k, kI_p). Properties of the Inverse-Wishart distribution give that that for $i = 1, \ldots, p$,

$$\text{var}(M_{ii}) = \frac{2k^2}{(k-p-1)^2(k-p-3)}. \tag{S.1}$$

Additionally, for $i, j = 1, \ldots, p$, where $j \neq i$

$$\text{var}(M_{ij}) = \frac{k^2(k-p-1)}{(k-p)(k-p-1)^2(k-p-3)}. \tag{S.2}$$

Finally we have that for $i, j = 1, \ldots, p$, $i \neq j$,

$$\text{cov}(M_{ij}, M_{ji}) = \frac{k^2(k-p-1)}{(k-p)(k-p-1)^2(k-p-3)}, \tag{S.3}$$

$$\text{cov}(M_{ii}, M_{jj}) = \frac{2k^2}{(k-p)(k-p-1)^2(k-p-3)}. \tag{S.4}$$

All other covariances $\text{cov}(M_{ij}, M_{br})$ are equal to zero unless they reduce to the cases in (S.3) or (S.4). Let $z = U^{\mathrm{T}}y$. Let $W = \text{cov}\,(MU^{\mathrm{T}}y) = \text{cov}(Mz)$. The elements of W can be determined using the properties in equations (S.1) to (S.4). Starting with the diagonal entries,

$$W_{ii} = \text{var}\left(\sum_{j=1}^{p} M_{ij}z_j\right) = \sum_{j=1}^{p} z_j^2 \text{var}(M_{ij}) + \sum_{j=1}^{p}\sum_{w\neq j}^{p} z_j z_w \text{cov}(M_{ij}, M_{iw}).$$

As $\text{cov}(M_{ij}, M_{iw})$ is equal to zero for all $w \neq j$ this simplifies to

$$W_{ii} = \text{var}\left(\sum_{j=1}^{p} M_{ij}z_j\right) = \sum_{j=1}^{p} z_j^2 \; \text{var}(M_{ij}).$$

It is helpful to split the sum into two pieces, a single term for $j = i$ and then a sum over the remaining indices. Grouping terms leads to an expression involving the model sum of squares MSS_F.

$$\begin{aligned}
W_{ii} &= z_i^2 \frac{2k^2}{(k-p-1)^2(k-p-3)} + \sum_{j=1, j\neq i}^{p} z_j^2 \frac{k^2(k-p-1)}{(k-p)(k-p-1)^2(k-p-3)} \\
&= z_i^2 \frac{2k^2(k-p)}{(k-p)(k-p-1)^2(k-p-3)} + \sum_{j=1, j\neq i}^{p} z_j^2 \frac{k^2(k-p-1)}{(k-p)(k-p-1)^2(k-p-3)} \\
&= z_i^2 \frac{2k^2(k-p-1) + 2k^2}{(k-p)(k-p-1)^2(k-p-3)} + \sum_{j=1, j\neq i}^{p} z_j^2 \frac{k^2(k-p-1)}{(k-p)(k-p-1)^2(k-p-3)} \\
&= \frac{k^2(k-p-1)}{(k-p)(k-p-1)^2(k-p-3)} \sum_{j=1}^{p} z_j^2 + \frac{k^2(k-p-1) + 2k^2}{(k-p)(k-p-1)^2(k-p-3)} z_i^2 \\
&= \frac{k^2(k-p-1)}{(k-p)(k-p-1)^2(k-p-3)} MSS_F + \frac{k^2(k-p+1)}{(k-p)(k-p-1)^2(k-p-3)} z_i^2.
\end{aligned}$$

In the second line the first term is modified to have the same denominator as the remainder sum. In the third line we add and subtract by $2k^2$ so that the numerator in the first term matches the

numerator in the remainder sum. This allows the z_j terms to be grouped into a sum over the full set of indexes $j = 1, \dots, p$ in the third line. The fourth line uses the fact that $\sum_{j=1}^{p} z_j^2 = z^{\mathrm{T}} z = y^{\mathrm{T}} U U^{\mathrm{T}} y = MSS_F$. For the off diagonal entries W_{ib} where $b \neq i$,

$$W_{ib} = \text{cov}\left(\sum_{j=1}^{p} M_{ij} z_j, \sum_{r=1}^{p} M_{br} z_r\right) = \sum_{j=1}^{p}\sum_{r=1}^{p} z_j z_r \text{ cov}(M_{ij}, M_{br}).$$

Now $\text{cov}(M_{ij}, M_{br})$ is only nonzero for $\text{cov}(M_{ib}, M_{bi})$ and $\text{cov}(M_{ii}, M_{bb})$. Using (S.3) and (S.4) we obtain

$$\begin{aligned} W_{ib} &= z_i z_b \text{ cov}(M_{ib}, M_{bi}) + z_i z_b \text{ cov}(M_{ii}, M_{bb}) \\ &= \frac{k^2(k-p-1)}{(k-p)(k-p-1)^2(k-p-3)} z_i z_b + \frac{2k^2}{(k-p)(k-p-1)^2(k-p-3)} z_i z_b \\ &= \frac{k^2(k-p+1)}{(k-p)(k-p-1)^2(k-p-3)} z_i z_b. \end{aligned}$$

The entire covariance matrix W can therefore be written compactly as

$$\begin{aligned} W &= \frac{k^2(k-p-1)}{(k-p)(k-p-1)^2(k-p-3)} (MSS_F I_p) + \frac{k^2(k-p+1)}{(k-p)(k-p-1)^2(k-p-3)} zz^T \\ &= \frac{k^2(k-p-1)}{(k-p)(k-p-1)^2(k-p-3)} \left(MSS_F I_p + \frac{(k-p+1)}{(k-p-1)} zz^T\right). \\ &= \frac{k^2}{(k-p)(k-p-1)(k-p-3)} \left(MSS_F I_p + \frac{(k-p+1)}{(k-p-1)} zz^T\right). \end{aligned}$$

Now $\beta_P = VD^{-1}Mz$. Therefore $\text{var}(\beta_P) = VD^{-1}\text{var}(Mz)D^{-1}V^{\mathrm{T}} = VD^{-1}WD^{-1}V^{\mathrm{T}}$. The variance of β_P is then a linear function of W,

$$\begin{aligned} \text{var}(\beta_P \mid y, X) &= VD^{-1}WD^{-1}V^{\mathrm{T}} \\ &= VD^{-1}\frac{k^2}{(k-p)(k-p-1)(k-p-3)} \left(MSS_F I_p + \frac{(k-p+1)}{(k-p-1)} zz^T\right) D^{-1}V^{\mathrm{T}} \\ &= \frac{k^2}{(k-p)(k-p-1)(k-p-3)} MSS_F (VD^{-2}V^{\mathrm{T}}) + \\ &\quad \frac{k^2(k-p+1)}{(k-p)(k-p-1)^2(k-p-3)} VD^{-1}zz^{\mathrm{T}}D^{-1}V^{\mathrm{T}}. \end{aligned} \quad \text{(S.5)}$$

Recall that $z = U^{\mathrm{T}} y$ and

$$\beta_F = (X^{\mathrm{T}}X)^{-1}X^{\mathrm{T}}y = VD^{-1}U^{\mathrm{T}}y = VD^{-1}z. \quad \text{(S.6)}$$

The term $VD^{-1}z$ appears in (S.5). Substituting (S.6) into (S.5) gives

$$\begin{aligned} \text{var}(\beta_P \mid y, X) = &\frac{k^2}{(k-p)(k-p-1)(k-p-3)} MSS_F (VD^{-2}V^{\mathrm{T}}) + \\ &\frac{k^2(k-p+1)}{(k-p)(k-p-1)^2(k-p-3)} \beta_F \beta_F^{\mathrm{T}}. \end{aligned}$$

A final simplification can be made by noting that $(X^{\mathrm{T}}X)^{-1} = VD^{-2}V^{\mathrm{T}}$ giving

$$\begin{aligned}\text{var}(\beta_P \mid y, X) &= \frac{k^2}{(k-p)(k-p-1)(k-p-3)} MSS_F (X^{\mathrm{T}}X)^{-1} + \frac{k^2(k-p+1)}{(k-p)(k-p-1)^2(k-p-3)}\beta_F\beta_F^{\mathrm{T}} \\ &= \frac{k^2}{(k-p)(k-p-1)(k-p-3)}\left(MSS_F(X^{\mathrm{T}}X)^{-1} + \frac{(k-p+1)}{(k-p-1)}\beta_F\beta_F^{\mathrm{T}}\right).\end{aligned}$$

The variance of $\beta_P^* = \{(k-p-1)/k\}\beta_P$ is then

$$\begin{aligned}\text{var}(\beta_P^* \mid y, X) &= \left(\frac{k-p-1}{k}\right)^2 \frac{k^2(k-p-1)}{(k-p)(k-p-1)^2(k-p-3)}\left(MSS_F(X^{\mathrm{T}}X)^{-1} + \frac{(k-p+1)}{(k-p-1)}\beta_F\beta_F^{\mathrm{T}}\right) \\ &= \frac{(k-p-1)}{(k-p)(k-p-3)}\left(MSS_F(X^{\mathrm{T}}X)^{-1} + \frac{(k-p+1)}{(k-p-1)}\beta_F\beta_F^{\mathrm{T}}\right). \qquad \text{(S.7)}\end{aligned}$$

3. RELATIVE EFFICIENCY OF COMPLETE AND PARTIAL SKETCHING

The relative efficiency of complete to partial sketching is

$$\begin{aligned}\frac{E_S(\|\beta_P^* - \beta_F\|_2^2 \mid y, X)}{E_S(\|\beta_S - \beta_F\|_2^2 \mid y, X)} &= \frac{\text{tr}\{\text{var}(\beta_P^*) \mid y, X\}}{\text{tr}\{\text{var}(\beta_S \mid y, X)\}} \\ &= \frac{(k-p-1)^2}{(k-p)(k-p-3)}\frac{MSS_F}{RSS_F}\frac{\text{tr}\{(X^{\mathrm{T}}X)^{-1}\}}{\text{tr}\{(X^{\mathrm{T}}X)^{-1}\}} + \\ &\quad \frac{(k-p-1)^2(k-p+1)}{(k-p)(k-p-3)(k-p-1)}\frac{\text{tr}(\beta_F\beta_F^{\mathrm{T}})}{RSS_F\text{tr}\{(X^{\mathrm{T}}X)^{-1}\}}. \qquad \text{(S.8)}\end{aligned}$$

It is easy to verify that

$$R_F^2 = \frac{MSS_F}{MSS_F + RSS_F} \implies \frac{R_F^2}{1-R_F^2} = \frac{MSS_F}{RSS_F}. \qquad \text{(S.9)}$$

Substituting (S.9) into (S.8) leads to

$$\begin{aligned}\frac{E_S(\|\beta_P^* - \beta_F\|_2^2 \mid y, X)}{E_S(\|\beta_S - \beta_F\|_2^2 \mid y, X)} &= \frac{(k-p-1)^2}{(k-p)(k-p-3)}\left(\frac{R_F^2}{1-R_F^2} + \frac{(k-p+1)}{(k-p-1)}\frac{\text{tr}(\beta_F\beta_F^{\mathrm{T}})}{RSS_F\text{tr}\{(X^{\mathrm{T}}X)^{-1}\}}\right) \\ &= \frac{(k-p-1)^2}{(k-p)(k-p-3)}\left(\frac{R_F^2}{1-R_F^2} + \frac{(k-p+1)}{(k-p-1)}\frac{\|\beta_F\|_2^2}{RSS_F\text{tr}\{(X^{\mathrm{T}}X)^{-1}\}}\right). \qquad \text{(S.10)}\end{aligned}$$

Now for $k > p+3$, $(k-p-1)^2/\{(k-p)(k-p-3)\}$ is greater than 1. Additionally, the final term in (S.10) always positive. This gives a lower bound

$$\frac{E_S(\|\beta_P^* - \beta_F\|_2^2 \mid y, X)}{E_S(\|\beta_S - \beta_F\|_2^2 \mid y, X)} \geq \frac{R_F^2}{1-R_F^2} + \frac{(k-p+1)}{(k-p-1)}\frac{\text{tr}(\beta_F\beta_F^{\mathrm{T}}))}{RSS_F\text{tr}\{(X^{\mathrm{T}}X)^{-1}\}} \geq \frac{R_F^2}{1-R_F^2}. \qquad \text{(S.11)}$$

For the upper bound, let the singular value decomposition of X be given by $X = UDV^{\mathrm{T}}$. It is simple to establish that $\beta_F = VD^{-1}U^{\mathrm{T}}y$ and $\|U^{\mathrm{T}}y\|_2^2 = MSS_F$. Using the Cauchy-Schwarz inequality

$$\frac{\|\beta_F\|_2^2}{RSS_F\text{tr}\{(X^{\mathrm{T}}X)^{-1}\}} \leq \frac{\|VD^{-1}\|_2^2\|U^{\mathrm{T}}y\|_2^2}{\text{tr}\{(X^{\mathrm{T}}X)^{-1}\}RSS_F} = \frac{\|VD^{-1}\|_2^2}{\text{tr}\{(X^{\mathrm{T}}X)^{-1}\}}\frac{MSS_F}{RSS_F} = \frac{\|VD^{-1}\|_2^2}{\text{tr}\{(X^{\mathrm{T}}X)^{-1}\}}\frac{R_F^2}{1-R_F^2}. \qquad \text{(S.12)}$$

Let $\sigma_i(X)$ refer to the i-th ordered singular value of X with $\sigma_1(X) \leq \sigma_2(X) \leq \ldots \leq \sigma_p(X)$. Then $\|VD^{-1}\|_2^2 = 1/(\sigma_1^2(X))$ and $\text{tr}\{(X^{\mathrm{T}}X)^{-1}\} = \sum_{i=1}^{p} 1/\sigma_i^2(X)$. As all the squared singular values are positive, this gives an upper bound on the quantity

$$\frac{\|VD^{-1}\|_2^2}{\text{tr}\{(X^{\mathrm{T}}X)^{-1}\}} = \frac{1/\sigma_1^2(X)}{\sum_{i=1}^{p} 1/\sigma_i^2(X)} \leq 1. \tag{S.13}$$

Substituting (S.13) into (S.12) gives an upper bound on the trailing term in terms of R_F^2:

$$\frac{\|\beta_F\|_2^2}{RSS_F \text{tr}\{(X^{\mathrm{T}}X)^{-1}\}} \leq \frac{\|VD^{-1}\|_2^2}{\text{tr}\{(X^{\mathrm{T}}X)^{-1}\}} \frac{R_F^2}{1-R_F^2} \leq \frac{R_F^2}{1-R_F^2}. \tag{S.14}$$

Substituting (S.14) into (S.10) leads to an upper bound on the relative efficiency

$$\begin{aligned}
\frac{E_S(\|\beta_P^* - \beta_F\|_2^2 \mid y, X)}{E_S(\|\beta_S - \beta_F\|_2^2 \mid y, X)} &= \frac{(k-p-1)^2}{(k-p)(k-p-3)} \left(\frac{R_F^2}{1-R_F^2} + \frac{(k-p+1)}{(k-p-1)} \frac{\|\beta_F\|_2^2}{RSS_F \text{tr}\{(X^{\mathrm{T}}X)^{-1}\}} \right) \\
&\leq \frac{(k-p-1)^2}{(k-p)(k-p-3)} \left(\frac{R_F^2}{1-R_F^2} + \frac{(k-p+1)}{(k-p-1)} \frac{R_F^2}{1-R_F^2} \right) \\
&= \frac{(k-p-1)^2}{(k-p)(k-p-3)} \left(\frac{2(k-p)}{(k-p-1)} \right) \frac{R_F^2}{1-R_F^2} \\
&= \frac{2(k-p-1)}{(k-p-3)} \frac{R_F^2}{1-R_F^2}. \qquad \text{(S.15)}
\end{aligned}$$

The relative efficiency bounds are given by (S.11) and (S.15).

4. COMBINED ESTIMATOR RESULTS

We first show that β_P^* and β_S are uncorrelated when y, X are treated as fixed, and the randomness is over the sketching matrix S. The covariance between β_P^* and β_S computed from the same sketch can be shown to be zero. Using the definition of covariance, and taking iterated expectations

$$\begin{aligned}
\text{cov}(\beta_P^*, \beta_S \mid y, X) &= E_S\left\{(\beta_P^* - \beta_F)(\beta_S - \beta_F)^{\mathrm{T}} \mid y, X\right\} \\
&= E_{\widetilde{X}}\left[E_{\widetilde{y}}\left\{(\beta_P^* - \beta_F)(\beta_S - \beta_F)^{\mathrm{T}} \mid \widetilde{X}, y, X\right\} \mid y, X\right]. \qquad \text{(S.16)}
\end{aligned}$$

Recall the hierarchical model for complete sketching,

$$\widetilde{y} \mid \widetilde{X}, y, X \sim N\left(\widetilde{X}\beta_F, \frac{RSS_F}{k} I_k\right).$$

Equivalently,

$$\widetilde{y} \mid \widetilde{X}, y, X = \widetilde{X}\beta_F + \widetilde{e},$$

where $\widetilde{e} \mid \widetilde{X} \sim N(0, k^{-1} RSS_F I_k)$. So

$$\beta_S \mid \widetilde{X}, y, X = \beta_F + (\widetilde{X}^{\mathrm{T}}\widetilde{X})^{-1}\widetilde{X}^{\mathrm{T}}\widetilde{e}. \tag{S.17}$$

Substituting (S.17) into (S.16),

$$\begin{aligned}\mathrm{cov}(\beta_P^*, \beta_S \mid y, X) &= E_{\widetilde{X}}\left[E_{\widetilde{e}\mid\widetilde{X}}\left\{(\beta_P^* - \beta_F)(\beta_F + (\widetilde{X}^{\mathrm{T}}\widetilde{X})^{-1}\widetilde{X}^{\mathrm{T}}\widetilde{e} - \beta_F)^{\mathrm{T}} \mid \widetilde{X}, y, X\right\} \mid y, X\right] \\ &= E_{\widetilde{X}}\left[\left\{(\beta_P^* - \beta_F)(\beta_F + (\widetilde{X}^{\mathrm{T}}\widetilde{X})^{-1}\widetilde{X}^{\mathrm{T}}E_{\widetilde{e}\mid\widetilde{X}}(\widetilde{e} \mid \widetilde{X}, y, X) - \beta_F)^{\mathrm{T}}\right\} \mid y, X\right] \\ &= E_{\widetilde{X}}\left[\{(\beta_P^* - \beta_F)(\beta_F - \beta_F)^{\mathrm{T}}\} \mid \widetilde{X}, y, X\right] \\ &= E_{\widetilde{X}}\left[\{(\beta_P^* - \beta_F)0^{\mathrm{T}}\} \mid \widetilde{X}, y, X\right] \\ &= 0_{p\times p}.\end{aligned}$$

Simple calculus shows that the value that minimises the expected mean squared error $E_S(\|\beta_C - \beta_F\|_2^2 \mid y, X)$ is

$$\phi_{\mathrm{opt}} = \frac{\mathrm{tr}(\mathrm{var}(\beta_P^* \mid y, X))}{\mathrm{tr}(\mathrm{var}(\beta_P^* \mid y, X)) + \mathrm{tr}(\mathrm{var}(\beta_S \mid y, X))}.$$

5. ONE-STEP CORRECTION

5.1. *Expected squared error*

The expected squared error for the one-step estimator is

$$\begin{aligned}E_S(\|\beta_H - \beta_F\|_2^2 \mid y, X) &= E_{\widetilde{X}}\left[E_{\widetilde{y}\mid\widetilde{X}}\left\{(\beta_S - \beta_F)^{\mathrm{T}}(I - \widetilde{H})^{\mathrm{T}}(I - \widetilde{H})(\beta_S - \beta_F) \mid y, X, \widetilde{X}\right\}\right] \\ &= E_{\widetilde{X}}\left[\mathrm{tr}\left(\mathrm{var}(\beta_S \mid y, X, \widetilde{X})(I - \widetilde{H})^{\mathrm{T}}(I - \widetilde{H})\right) \mid y, X\right] \\ &= E_{\widetilde{X}}\left[\mathrm{tr}\left(\frac{RSS_F}{k}(\widetilde{X}^{\mathrm{T}}\widetilde{X})^{-1}(I - \widetilde{H})^{\mathrm{T}}(I - \widetilde{H})\right) \mid y, X\right]. \qquad \text{(S.18)}\end{aligned}$$

Now $(\widetilde{X}^{\mathrm{T}}\widetilde{X})^{-1} \mid y, X \sim$ Inverse-Wishart$(k, k(X^{\mathrm{T}}X)^{-1})$. Assuming that $k \geq p$, $\widetilde{X}$ is invertible with probability one, and

$$(\widetilde{X}^{\mathrm{T}}\widetilde{X})^{-1} = VD^{-1}(U^{\mathrm{T}}S^{\mathrm{T}}SU)^{-1}D^{-1}V^{\mathrm{T}},$$

where $X = UDV^{\mathrm{T}}$ is the SVD of X. The random matrix $\widetilde{H} = (\widetilde{X}^{\mathrm{T}}\widetilde{X})^{-1}X^{\mathrm{T}}X$ can then be written as

$$\begin{aligned}\widetilde{H} &= VD^{-1}(U^{\mathrm{T}}S^{\mathrm{T}}SU)^{-1}D^{-1}V^{\mathrm{T}}X^{\mathrm{T}}X \\ &= VD^{-1}(U^{\mathrm{T}}S^{\mathrm{T}}SU)^{-1}D^{-1}V^{\mathrm{T}}VD^2V^{\mathrm{T}} \\ &= VD^{-1}(U^{\mathrm{T}}S^{\mathrm{T}}SU)^{-1}DV^{\mathrm{T}}. \qquad \text{(S.19)}\end{aligned}$$

Using (S.19) in the expansion of (S.18)

$$\begin{aligned}E_S(\|\beta_H - \beta_F\|_2^2 \mid y, X) &= \frac{RSS_F}{k}E_{\widetilde{X}}\left[\mathrm{tr}\left((\widetilde{X}^{\mathrm{T}}\widetilde{X})^{-1}(I - \widetilde{H})^{\mathrm{T}}(I - \widetilde{H})\right) \mid y, X\right] \\ &= \frac{RSS_F}{k}E_{\widetilde{X}}\left[\mathrm{tr}\left((\widetilde{X}^{\mathrm{T}}\widetilde{X})^{-1}(I - 2\widetilde{H}^{\mathrm{T}} + \widetilde{H}^{\mathrm{T}}\widetilde{H})\right) \mid y, X\right] \\ &= \frac{RSS_F}{k}E_{\widetilde{X}}\left[\mathrm{tr}\left\{(\widetilde{X}^{\mathrm{T}}\widetilde{X})^{-1} - 2(\widetilde{X}^{\mathrm{T}}\widetilde{X})^{-1}\widetilde{H}^{\mathrm{T}} + (\widetilde{X}^{\mathrm{T}}\widetilde{X})^{-1}\widetilde{H}^{\mathrm{T}}\widetilde{H}\right\} \mid y, X\right] \\ &= \frac{RSS_F}{k}E_{\widetilde{X}}\left[\mathrm{tr}(\widetilde{X}^{\mathrm{T}}\widetilde{X})^{-1} - 2\mathrm{tr}\{(\widetilde{X}^{\mathrm{T}}\widetilde{X})^{-1}\widetilde{H}^{\mathrm{T}}\} + \mathrm{tr}\{\widetilde{H}(\widetilde{X}^{\mathrm{T}}\widetilde{X})^{-1}\widetilde{H}^{\mathrm{T}}\} \mid y, X\right]. \qquad \text{(S.20)}\end{aligned}$$

Now

$$\begin{aligned}\mathrm{tr}\{(\widetilde{X}^{\mathrm{T}}\widetilde{X})^{-1}\widetilde{H}^{\mathrm{T}}\} &= \mathrm{tr}\{VD^{-1}(U^{\mathrm{T}}S^{\mathrm{T}}SU)^{-1}D^{-1}V^{\mathrm{T}}VD(U^{\mathrm{T}}S^{\mathrm{T}}SU)^{-1}D^{-1}V^{\mathrm{T}}\}\\ &= \mathrm{tr}\{VD^{-1}(U^{\mathrm{T}}S^{\mathrm{T}}SU)^{-2}D^{-1}V^{\mathrm{T}}\}\\ &= \mathrm{tr}\{(U^{\mathrm{T}}S^{\mathrm{T}}SU)^{-2}D^{-2}\}, \qquad (S.21)\end{aligned}$$

and

$$\begin{aligned}\mathrm{tr}\{\widetilde{H}(\widetilde{X}^{\mathrm{T}}\widetilde{X})^{-1}\widetilde{H}^{\mathrm{T}}\} &= \mathrm{tr}\{VD^{-1}(U^{\mathrm{T}}S^{\mathrm{T}}SU)^{-1}DV^{\mathrm{T}}VD^{-1}(U^{\mathrm{T}}S^{\mathrm{T}}SU)^{-2}D^{-1}V^{\mathrm{T}}\}\\ &= \mathrm{tr}\{(U^{\mathrm{T}}S^{\mathrm{T}}SU)^{-3}D^{-2}\}. \qquad (S.22)\end{aligned}$$

Let $\widetilde{W} = (U^{\mathrm{T}}S^{\mathrm{T}}SU)$. Substituting (S.21) and (S.22) into (S.20)

$$\begin{aligned}E_S(\|\beta_H - \beta_F\|_2^2 \mid y, X) &= \frac{RSS_F}{k} E_{\widetilde{X}}\left(\mathrm{tr}\{\widetilde{W}^{-1}D^{-2}\} - 2\mathrm{tr}\{\widetilde{W}^{-2}D^{-2}\} + \mathrm{tr}\{\widetilde{W}^{-3}D^{-2}\} \mid y, X\right)\\ &= \frac{RSS_F}{k}\Big(\mathrm{tr}\{E_{\widetilde{X}}(\widetilde{W}^{-1})D^{-2}\} - 2\mathrm{tr}\{E_{\widetilde{X}}(\widetilde{W}^{-2})D^{-2}\} +\\ &\qquad \mathrm{tr}\{E_{\widetilde{X}}(\widetilde{W}^{-3})D^{-2}\} \mid y, X\Big).\end{aligned}$$

The required moments can be computed using the fact that $\widetilde{W}^{-1} \mid X, y \sim$ Inverse-Wishart(k, kI_p). From results in Letac & Massam (2004), the required moments can be expressed as

$$E_{\widetilde{X}}(\widetilde{W}^{-1} \mid y, X) = c_1 I_p, \quad E_{\widetilde{X}}(\widetilde{W}^{-2} \mid y, X) = c_2 I_p, \quad E_{\widetilde{X}}(\widetilde{W}^{-3} \mid y, X) = c_3 I_p, \qquad (S.23)$$

for scalars c_1, c_2, c_3 that are functions of k and p. The expected squared error for β_H is

$$E_S(\|\beta_H - \beta_F\|_2^2 \mid y, X) = \frac{RSS_F}{k}(c_1 - 2c_2 + c_3)\mathrm{tr}(D^{-2}). \qquad (S.24)$$

The expected squared error for β_S can be written as

$$E_S(\|\beta_S - \beta_F\|_2^2 \mid y, X) = \frac{RSS_F}{k-p-1}\mathrm{tr}\{(X^{\mathrm{T}}X)^{-1}\} = \frac{RSS_F}{k-p-1}\mathrm{tr}(D^{-2}). \qquad (S.25)$$

Dividing (S.24) by (S.25), the relative efficiency of β_S to β_H is given by

$$\frac{E_S(\|\beta_H - \beta_F\|_2^2 \mid y, X)}{E_S(\|\beta_S - \beta_F\|_2^2 \mid y, X)} = \frac{k-p-1}{k}(c_1 - 2c_2 + c_3). \qquad (S.26)$$

From results on page 308 of Letac & Massam (2004), c_1 and c_2 in (S.23) can be expressed as

$$\begin{aligned}c_1 &= \frac{k}{k-p-1},\\ c_2 &= \frac{k^2 p}{(k-p)(k-p-1)(k-p-3)} + \frac{k^2}{(k-p)(k-p-3)}.\end{aligned}$$

Let $a_3 = (k-p+1)(k-p)(k-p-1)(k-p-3)(k-p-5)$. By equation (44) in Letac & Massam (2004) the constant c_3 in (S.23) can be expressed as

$$c_3 = \frac{2(k^3p^2)}{a_3} + \frac{3(k-p-1)(pk^3)}{a_3} + \frac{(k-p-1)^2k^3}{a_3}.$$

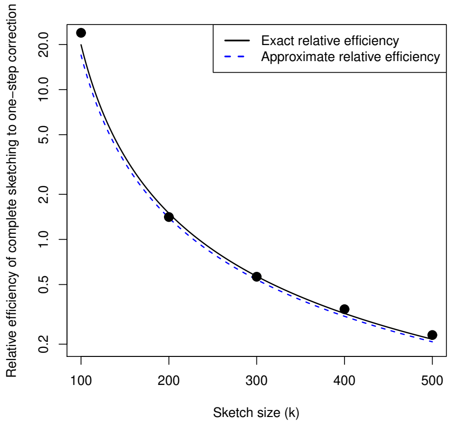

Fig. 1. Relative efficiency of β_S compared to β_H for $p = 50$. The y-axis is on a log-scale. The black points show simulation results discussed in Section 5.2

Let ω denote the variable to sketch size ratio $\omega = p/k$. Substituting $k - p = k(1 - \omega)$ into the expressions for c_1, c_2, c_3 and dropping constants, the moments can be approximated as

$$c_1' = \frac{1}{1-\omega}, \quad c_2' = \frac{\omega}{(1-\omega)^3} + \frac{1}{(1-\omega)^2}, \quad c_3' = \frac{2\omega^2}{(1-\omega)^5} + \frac{3\omega(1-\omega)}{(1-\omega)^5} + \frac{1}{(1-\omega)^3}.$$

For fixed p, $\lim_{k\to\infty} c_1'/c_1 = 1$, $\lim_{k\to\infty} c_2'/c_2 = 1$ and $\lim_{k\to\infty} c_3'/c_3 = 1$. For large k, we have an approximation for the relative efficiency

$$E_S(\|\beta_H - \beta_F\|_2^2 \mid y, X) \approx (1-\omega)(c_1' - 2c_2' + c_3'). \qquad \text{(S.27)}$$

Figure 1 compares the asymptotic approximation (S.27) of the relative efficiency (dashed blue line) to the exact expression (S.26) (solid black line) for $p = 50$ over a range of values for the sketch size k. The asymptotic approximation gives a slight underestimate.

5.2. *Synthetic data*

To assess the benefit of one-step correction we generated a synthetic dataset with $n = 10000$, $p = 50$ and an R_F^2 of close to 0.5. The dataset consisted of responses y_i and covariates x_i, $(i = 1, \ldots, n)$. Covariates x_i were drawn from a multivariate normal distribution with mean zero and covariance matrix Σ, with elements $\Sigma_{ij} = 0.5^{|i-j|}$. Responses were simulated independently using the standard linear model $y_i = x_i^{\mathrm{T}}\beta_0 + \epsilon_i$ $(i = 1, \ldots, n)$, where ϵ_i is a distributed as $N(0, 0.45)$. Each element of β_0 was sampled independently from a $N(0, 0.01)$ distribution. We compared the single pass estimators β_S, β_P^* to the combined estimator β_C with the optimal weight ϕ_{opt}, and the one-step estimator β_H. We applied the Gaussian, Hadamard, Clarkson-Woodruff and uniform subsampling sketches. We computed one hundred sketches at a range of sketch sizes k. We calculated the conditional sketching error $\|\widehat{\beta} - \beta_F\|_2^2$ for each sketched estimator $\widehat{\beta}$ in each replicate. Figure 2 plots the average error for the estimators β_S, β_P^*, β_C and β_H against the sketch size k. As expected, the combined estimator β_C has a mean squared error that is roughly half that of β_S or β_P^* at all sketch sizes k. When k/p is small, the one-step estimator β_H has a higher mean squared error than the single pass estimator β_S. As the ratio k/p increases, the one-step estimator β_H becomes more efficient than the weighted estimator β_C.

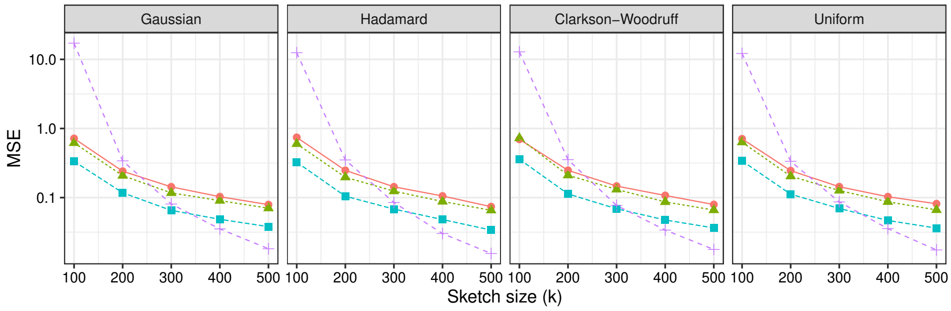

Fig. 2. Comparison of sketching estimators on synthetic dataset with $R_F^2 \approx 0.5$. The y-axis is on a log scale. The average squared error for the sketching estimator is plotted against sketch size. Results are shown for β_S ($\circ$), β_P^* ($\triangle$), the weighted combined estimator β_C ($\square$) and the one-step estimator β_H ($+$).

The results are similar for each of the data-oblivious projections, suggesting that the asymptotic approximations are reasonable for this dataset.

The black points in Figure 1 show the simulated relative efficiencies of β_S to β_H using the Gaussian sketch. There is high agreement between the empirical results and the predictions using (S.26) and (S.27).

6. Proof of Theorem 2

Standard tools including Lindeberg's condition, Lyapunov's condition, the Cramér-Wold device and the continuous mapping theorem are all used in the proof, see Van Der Vaart (1998) for definitions and proofs of these results. We will rely on a central limit theorem for jointly symmetric, pairwise independent random variables (Pruss & Szynal, 2000). A collection of random variables $(Z_1, \ldots Z_n)$ is said to be jointly symmetric if $(Z_1, \ldots Z_n)$ has the same distribution as $(q_1 Z_1, \ldots, q_n Z_n)$, where $q_i \in \{+1, -1\}$ for $i = 1, \ldots, n$. Given a set of random variables $Y_1, \ldots, Y_n$, a jointly symmetric collection $Z_1, \ldots, Z_n$ can be formed by sampling n independent Rademacher random variables $h_1, \ldots, h_n$, and setting $Z_i = h_i Y_i$ (Pruss & Szynal, 2000). It is possible to establish a central limit theorem for jointly symmetric, pairwise independent random variables.

Theorem S.1 (Pruss & Szynal (2000), Theorem 1, Corollary 2). *For each $n \in \mathbb{N}$, let $Z_{n1}, Z_{n2}, \ldots, Z_{nr_n}$ be a sequence of jointly symmetric pairwise independent random variables with $E(Z_{ni}) = 0$ and $\mathrm{var}(Z_{ni}) = \sigma_{ni}^2$ for $i = 1, \ldots, r_n$. Let $s_n^2 = \sum_{i=1}^{r_n} \sigma_{ni}^2$ and assume that $r_n \to \infty$ as $n \to \infty$. Suppose the triangular array of random variables satisfies Lindeberg's condition. Then as $n \to \infty$, $s_n^{-1} \sum_{i=1}^{r_n} Z_{ni}$ converges in distribution to $N(0, 1)$.*

Not all triangular arrays with pairwise independent random variables in each row satisfy a central limit theorem. The joint symmetry property is very important (Pruss & Szynal, 2000; Svante, 1988).

To use Theorem S.1 we need to verify that Lindeberg's condition is satisfied. This can be hard to show directly. Lyapunov's condition implies Linderberg's condition and is often easier to establish. Billingsley (1999) gives a useful lemma for triangular arrays of random variables to satisfy Lyapunov's condition.

Theorem S.2 (Billingsley, 1999). *For each $n \in \mathbb{N}$ let $Z_{n1}, Z_{n2}, \ldots, Z_{nr_n}$ be a sequence of random variables with $E[Z_{ni}] = 0$ and $var(Z_{ni}^2) = \sigma_{ni}^2$ for $i = 1, \ldots, r_n$. Define*

$s_n^2 = \sum_{i=1}^{r_n} \sigma_{ni}^2$ each n. Suppose that the rows of the triangular array are standardised such that $s_n^2 = 1$ for all n. Suppose that $r_n \to \infty$ as $n \to \infty$. Suppose we have a sequence of upper bounds (Z_n) such that $|Z_{ni}| \le K_n$ almost surely for all $i = 1, \ldots, r_n$. Then a sufficient condition for Lyapnuov's condition to hold is $K_n \to 0$ as $n \to \infty$.

If the triangular array of random variables can be appropriately bounded, we can use Theorem S.2 to show that Lyapunov's condition holds, and subsequently that Lindeberg's condition holds.

The outline of the proof is to first use the Cramér-Wold device to reduce the problem of multivariate convergence to univariate convergence. We then form a triangular array of random variables such that elements in each row are jointly symmetric and pairwise independent. We then show that triangular array satisfies Lyapunov's condition using Theorem S.2 (thus implying Lindeberg's condition). Assumption 1 on the maximum leverage score enforces the necessary cap on the rate of growth. Theorem S.1 is then used to establish asymptotic normality.

Let $n \in \mathbb{N}$ index the sequence of source datasets of increasing size. We assume that the source dataset consists of r_n observations where $r_n \to \infty$ as $n \to \infty$. For now we can take take $r_n = n$ to ease interpretation. We take the singular value decomposition of each dataset $A_{(n)} = U_{(n)} D_{(n)} V_{(n)}^{\mathrm{T}}$. All results in this section treat the source dataset $A_{(n)}$ as fixed, only the sketching matrix is random. We consider the sequence of whitened sketched datasets

$$\widetilde{A} V_{(n)} D_{(n)}^{-1} = (SA) V_{(n)} D_{(n)}^{-1} = S U_{(n)} D_{(n)} V_{(n)}^{\mathrm{T}} V_{(n)} D_{(n)}^{-1} = S U_{(n)}.$$

The whitened sketched dataset $\widetilde{A} V_{(n)} D_{(n)}^{-1}$ has a $MN(0, I_k, I_d/k)$ distribution when S is a Gaussian sketch. We need to show that as n tends to infinity, $SU_{(n)}$ convergences in distribution to a $MN(0, I_k, I_d/k)$ random matrix for both the Clarkson-Woodruff and Hadamard sketches.

Let $u_{(n)i}^{\mathrm{T}}$ denote row i of the matrix of left singular vectors $U_{(n)}$. An important property is that for all n, the sum of the norms of the leverage scores always equals the number of variables in the source dataset d.

$$\sum_{i=1}^{r_n} \|u_{(n)i}\|_2^2 = d. \tag{S.28}$$

As n increases, the typical norm of each vector $u_{(n)i}$, $i \in \{1, \ldots, r_n\}$ is expected to decrease. The standardisation property in equation (S.28), namely that $\sum_{i=1}^{r_n} \|u_{(n)i}\|_2^2 = d$ for all n is similar to the assumption that $s_n = 1$ in each row of the triangular array of random variables in Theorem S.2. Assumption 1 on the leverage scores, where the maximum individual norm tends to zero is similar to the assumption that $K_n \to 0$ in Theorem S.2. This will be made more explicit in the proofs.

As indicated we use the Cramér-Wold device. Let z_n represent the kd length vector formed by stacking transposed rows of the whitened sketched dataset $\widetilde{U} = SU_{(n)}$. Let $\widetilde{u}_j^{\mathrm{T}}$ give row j in $\widetilde{U}$ for $j = 1, \ldots, k$. Formally,

$$z_n = \begin{pmatrix} \widetilde{u}_1 \\ \widetilde{u}_2 \\ \vdots \\ \widetilde{u}_k \end{pmatrix}. \tag{S.29}$$

Let us define the random matrix $k \times d$ random matrix W as having the matrix normal distribution $W \sim MN(0, I_k, I_d/k)$. Let w_i^{T} refer to row i in W for $i = 1, \ldots, k$. Let z_L refer to the stacked

transposed rows of W, so

$$z_L = \begin{pmatrix} w_1 \\ w_2 \\ \vdots \\ w_k \end{pmatrix}. \tag{S.30}$$

Let λ be an arbitrary unit vector in $\mathbb{R}^{k \times d}$. It will be useful to also partition the vector λ into k sub-vectors,

$$\lambda = \begin{pmatrix} \lambda_1 \\ \lambda_2 \\ \vdots \\ \lambda_k \end{pmatrix}, \tag{S.31}$$

where λ_j is a d-dimensional vector for $j = 1, \ldots, k$. For any unit vector $\lambda \in \mathbb{R}^{k \times d}$, $\lambda^{\mathrm{T}} z_L$ is distributed as $N(0, 1/k)$. Using the Cramér-Wold device, to show that the distribution of the whitened sketched data $SA_{(n)}V_{(n)}D_{(n)}^{-1}$ converges to that of W, it is sufficient to show that for any fixed $k \times d$ length unit vector λ, $\lambda^{\mathrm{T}} z_n$ converges in distribution to $N(0, 1/k)$ as $n \to \infty$.

6.1. *Clarkson-Woodruff sketch*

The Clarkson-Woodruff sketch can be represented as the product of two independent random matrices, $S = \Gamma D$, where Γ is a random $k \times n$ matrix and D is a random $n \times n$ matrix. The diagonal matrix D contains n independent Rademacher random variables on the diagonal. Let $h_i \in \{+1, -1\}$ be the random sign in element D_{ii}. The matrix Γ is formed by choosing one element in each column independently and setting the entry to $+1$. Element Γ_{ij} is equal to $+1$ if we add observation i in the original dataset to sketched observation j. The signs in row i are flipped if h_i is equal to negative one. Each observation in the original dataset is assigned to one sketched observation as each column of Γ contains a single $+1$ entry. Using a Clarkson-Woodruff sketch row j in the sketched data matrix can be represented as

$$\widetilde{u}_j^{\mathrm{T}} = \sum_{i=1}^{n} h_i \Gamma_{ij} u_{(n)i}^{\mathrm{T}},$$

where h_i represents the random sign flip applied to row i of the original data matrix, and Γ_{ij} is the indicator variable which is equal to one if row i of the original data is added to row j of the sketched dataset.

Let us consider the linear combination $\lambda^{\mathrm{T}} z$, where λ and z are defined as in (S.29) and (S.31) respectively. The sum over the k rows in the sketched dataset can be rearranged into a sum over the n rows in the source dataset,

$$\lambda^{\mathrm{T}} z_n = \sum_{j=1}^{k} \lambda_j^{\mathrm{T}} \widetilde{u}_j = \sum_{j=1}^{k} \lambda_j^{\mathrm{T}} \sum_{i=1}^{n} h_i \Gamma_{ij} u_{(n)i} = \sum_{i=1}^{n} h_i \sum_{j=1}^{k} \Gamma_{ij} \lambda_j^{\mathrm{T}} u_{(n)i}. \tag{S.32}$$

The scalar $\lambda^{\mathrm{T}} z_n$ is equal to the sum of n independent random variables. Independence holds as the signs flips h_i on each observation are independent, and each column of Γ is independent.

In the language of Theorem S.2 we can form a triangular array of random variables setting

$$Z_{ni} = h_i \sum_{j=1}^{k} \Gamma_{ij} \lambda_j^{\mathrm{T}} u_{(n)i}. \tag{S.33}$$

for $i = 1, \ldots, n$ and $n \in \mathbb{N}$. The linear combination in (S.32) then be expressed as a row sum over the triangular array defined in (S.33):

$$\lambda^{\mathrm{T}} z_n = \sum_{i=1}^{n} Z_{ni}. \tag{S.34}$$

Our goal of showing that $\lambda^{\mathrm{T}} z_n$ converges in distribution to a $N(0, 1/k)$ random variable is achieved if we can show that $\sum_{i=1}^{n} Z_{ni}$ converges in distribution to a $N(0, 1/k)$ random variable.

It is worth making a connection to Theorem S.1, because of the random sign flips h_i appearing in (S.33), we have a sequence of mutually independent jointly symmetric random variables. Theorem S.1 can be used to establish asymptotic normality of the sum in (S.34) and hence the linear combination $\lambda^{\mathrm{T}} z_n$. To show that the triangular array of random variables defined in (S.33) satisfies Lindeberg's condition we use Theorem S.2. Set $s_n^2 = \sum_{i=1}^{n} \mathrm{var}(Z_{ni})$. We first determine s_n^2. We then form the necessary sequence of upper bounds K_n such that $|Z_{ni}| \leq K_n$ almost surely for $i = 1, \ldots, n$. The variance of a single term in the sum (S.32) is

$$\mathrm{var}(Z_{ni}) = \mathrm{var}\left(h_i \sum_{j=1}^{k} \Gamma_{ij} \lambda_j^{\mathrm{T}} u_{(n)i} \right) = \sum_{j=1}^{k} \frac{1}{k} \lambda_j^{\mathrm{T}} u_{(n)i} u_{(n)i}^{\mathrm{T}} \lambda_j. \tag{S.35}$$

The row-wise variance totals s_n^2 are then

$$\begin{aligned}
s_n^2 &= \sum_{i=1}^{n} \mathrm{var}(Z_{ni}) \\
&= \sum_{i=1}^{n} \mathrm{var}\left(h_i \sum_{j=1}^{k} \Gamma_{ij} \lambda_j^{\mathrm{T}} u_{(n)i} \right) \\
&= \frac{1}{k} \sum_{i=1}^{n} \sum_{j=1}^{k} \lambda_j^{\mathrm{T}} u_{(n)i} u_{(n)i}^{\mathrm{T}} \lambda_j \\
&= \frac{1}{k} \sum_{j=1}^{k} \lambda_j^{\mathrm{T}} \left(\sum_{i=1}^{n} u_{(n)i} u_{(n)i}^{\mathrm{T}} \right) \lambda_j \\
&= \frac{1}{k} \sum_{j=1}^{k} \lambda_j^{\mathrm{T}} U_{(n)}^{\mathrm{T}} U_{(n)} \lambda_j \\
&= \frac{1}{k} \sum_{j=1}^{k} \lambda_j^{\mathrm{T}} \lambda_j \\
&= \frac{1}{k}.
\end{aligned}$$

The fact that $U_{(n)}^{\mathrm{T}} U_{(n)} = I_d$ for all n serves as a useful normalisation to give stable limiting behaviour. The step in the last line follows as we have taken λ to be a unit vector. We have

$s_n^2 = 1/k$ for all n in the triangular array. We now establish a sequence of upper bounds (K_n). As the random variables in the construction of construction of the sketch are bounded, we can bound the random variables in the triangular array using the leverage scores of the sequence of source datasets. Now as the random sign $h_i \in \{+1, -1\}$,

$$|Z_{ni}| = |h_i \sum_{j=1}^{k} \Gamma_{ij} \lambda_j^{\mathrm{T}} u_{(n)i}| = |\left(\sum_{j=1}^{k} \Gamma_{ij} \lambda_j^{\mathrm{T}}\right) u_{(n)i}|. \tag{S.36}$$

By the Cauchy-Schwarz inequality

$$|\left(\sum_{j=1}^{k} \Gamma_{ij} \lambda_j^{\mathrm{T}}\right) u_{(n)i}| \leq \|\sum_{j=1}^{k} \Gamma_{ij} \lambda_j\|_2 \|u_{(n)i}\|_2. \tag{S.37}$$

As $\Gamma_{ij} = 1$ for a single $j \in \{1, \ldots, k\}$ and is zero otherwise we have that

$$\|\sum_{j=1}^{k} \Gamma_{ij} \lambda_j\|_2 \leq \max_{j=1,\ldots,k} \|\lambda_j\|_2 \leq 1. \tag{S.38}$$

The last inequality follows as λ is a unit vector. Substituting (S.38) and (S.37) into (S.36) leads to the bound $|Z_{ni}| \leq \|u_{(n)i}\|_2$. We can then form the sequence of upper bounds K_n,

$$K_n = \max_{i=1,\ldots,n} \|u_{(n)i}\|_2.$$

We have that $|Z_{ni}| \leq K_n$ almost surely for $i = 1, \ldots, n$ and $n \in \mathbb{N}$. Using Assumption 1 on the limiting behaviour of the leverage scores $\lim_{n\to\infty} K_n = \lim_{n\to\infty} \max_{i=1,\ldots,n} \|u_{(n)i}\|_2 = 0$. By theorem S.2 we have that the triangular array of random variables in (S.33) satisfies Lindeberg's condition. As such the conditions of Theorem S.1 are satisfied, giving that $\lambda^{\mathrm{T}} z_n$ converges in distribution to $N(0, 1/k)$. Finally, the Cramér-Wold device gives that the whitened sketched dataset has a limiting matrix normal distribution, that is $\widetilde{A} V_{(n)} D_{(n)}^{-1}$ converges in distribution to a $MN\left(0, I_k, I_d/k\right)$ random matrix.

6.2. *Hadamard sketch*

Recall that the Hadamard sketch is defined through $S = \Phi H D/\sqrt{k}$. Here H is a Hadamard matrix. Hadamard matrices are square matrices with 2^n rows for some integer n. To take limits we have to define our sequence of source datasets $(A_{(n)} = U_{(n)} D_{(n)} V_{(n)}^{\mathrm{T}})$ as having $r_n = 2^n$ rows for $n \in \mathbb{N}^+$. In practice when taking a Hadamard sketch we pad the original dataset with zeros if the original number of observations is not a power of two. To rigourously establish asymptotic normality for the Hadamard sketch we have to take $r_n = 2^n$. The indexing change to $r_n = 2^n$ instead of $r_n = n$ has very little impact on the underlying arguments.

There are two independent sources of randomness in a Hadamard sketch, the $r_n = 2^n$ independent random Rademacher variables in the diagonal matrix D, and the random matrix Φ which subsamples k rows with replacement from the Hadamard matrix H. Hadamard matrices have a number of properties that we will use (Anderson, 1997, section 3.2).

- (P1) The first column contains all ones.
- (P2) Every column other than the first contains an equal number of $+1$ and -1 entries.
- (P3) Consider any two different columns i and s, where $i, s \in \{2, \ldots, r_n\}$, $i \neq s$. Columns i and s will have $+1$ together in a quarter of the rows, and -1 together in a quarter of the rows.

Furthermore, a quarter of the rows will have $+1$ in column i and -1 in column s. Similarly, a quarter of the rows will have -1 in column i and $+1$ in column s.

Let M represent the random $k \times n$ matrix from the subsampling operation $M = \Phi H$. Let m_{ji} refer to the element in row j and column i of M. Each element in M is equal to $+1$ or -1. Let $h_i \in \{+1, -1\}$ be the random sign in element D_{ii}. We now represent the Hadamard sketch as $S = MD/\sqrt{k}$.

The structure of the Hadamard matrix gives the random matrix M some useful properties. Consider an arbitrary row j in M. By (P1) listed above regarding the first column of M, $m_{j1} = 1$ with probability one. For the other columns, $m_{ji} = 1$ with probability half, and $m_{ji} = -1$ with probability half for $i = 2, \dots, r_n$ by (P2). By (P3) listed above, we have pairwise independence between elements in row j of M, that is $p(m_{ji}|m_{js}) = p(m_{ji})$ for $i, s \in \{1, \dots, r_n\}$, $i \neq s$. As rows of M are sampled independently, each column of M is pairwise independent.

Row j in the sketched dataset is given by

$$\widetilde{u}_j^{\mathrm{T}} = \frac{1}{\sqrt{k}} \sum_{i=1}^{r_n} m_{ji} h_i u_{(n)i}^{\mathrm{T}}.$$

Let us again consider the linear combination $\lambda^{\mathrm{T}} z_n$, where λ and z_n are defined as in (S.29) and (S.31) respectively. The sum over the k rows in the sketched dataset can be rearranged into a sum over the $r_n = 2^n$ rows in the source dataset,

$$\lambda^{\mathrm{T}} z_n = \sum_{j=1}^{k} \lambda_j^{\mathrm{T}} \widetilde{u}_j = \frac{1}{\sqrt{k}} \sum_{j=1}^{k} \lambda_j^{\mathrm{T}} \sum_{i=1}^{r_n} m_{ji} h_i u_{(n)i} = \frac{1}{\sqrt{k}} \sum_{i=1}^{r_n} h_i \left(\sum_{j=1}^{k} m_{ji} \lambda_j^{\mathrm{T}} \right) u_{(n)i}. \quad \text{(S.39)}$$

In the language of Theorem S.2 we can form a triangular array of random variables setting

$$Z_{ni} = \frac{1}{\sqrt{k}} h_i \left(\sum_{j=1}^{k} m_{ji} \lambda_j^{\mathrm{T}} \right) u_{(n)i}. \quad \text{(S.40)}$$

for $i = 1, \dots, r_n$ and $n \in \mathbb{N}$. The linear combination in (S.39) can then be expressed as a row sum of the triangular array defined by (S.40)

$$\lambda^{\mathrm{T}} z_n = \sum_{i=1}^{r_n} Z_{ni}. \quad \text{(S.41)}$$

Our goal of showing that $\lambda^{\mathrm{T}} z_n$ converges in distribution to a $N(0, 1/k)$ random variable is achieved if we can show that $\sum_{i=1}^{r_n} Z_{ni}$ converges in distribution to a $N(0, 1/k)$ random variable.

The sequence of random variables in each row of the triangular array $Z_{n1}, \dots, Z_{nr_n}$ are not mutually independent over $i = 1 \dots, r_n$. This is because the columns of M are not mutually independent. However, as the columns of M are pairwise independent, the random sums $\sum_{j=1}^{k} m_{ji} \lambda_j^{\mathrm{T}}$ appearing in (S.40) are also pairwise independent. Again making a connection to Theorem S.1, the independent sign flips h_i appearing in (S.40) ensure that the random variables in each row of the triangular array are jointly symmetric and pairwise independent.

Theorem S.1 can be used to establish asymptotic normality of the sum in (S.41) and hence the linear combination $\lambda^{\mathrm{T}} z_n$. To show that the triangular array of random variables defined in (S.40) satisfies Lindeberg's condition we use Theorem S.2. Set $s_n^2 = \sum_{i=1}^{r_n} \mathrm{var}(Z_{ni})$. We first determine s_n^2. We then form the necessary sequence of upper bounds K_n such that $|Z_{ni}| \leq K_n$ almost surely for $i = 1, \dots, r_n$.

We start by considering the variance of a single term in the triangular array $\text{var}(Z_{ni})$. We have that

$$\text{var}(Z_{ni}) = \frac{1}{k}\text{var}\left(h_i\left(\sum_{j=1}^{k} m_{ji}\lambda_j^{\mathrm{T}}\right)u_{(n)i}\right) \tag{S.42}$$

It is important to consider the covariance between the elements of the sum over $j = 1, \ldots, k$. For $i \neq 1$ and $j, v \in \{1, \ldots, k\}$, $j \neq v$ the covariance is zero

$$\begin{aligned}\text{cov}\left(h_i m_{ji}\lambda_j^{\mathrm{T}}u_{(n)i}, h_i m_{vi}\lambda_v^{\mathrm{T}}u_{(n)i}\right) &= E\left[h_i^2 m_{ji}m_{vi}\lambda_j^{\mathrm{T}}u_{(n)i}\lambda_v^{\mathrm{T}}u_{(n)i}\right]\\ &= E\left[m_{ji}m_{vi}\right]\lambda_j^{\mathrm{T}}u_{(n)i}\lambda_v^{\mathrm{T}}u_{(n)i}\\ &= 0.\end{aligned}$$

We use (P2) to conclude that $E\left[m_{ji}m_{vi}\right] = 0$. Therefore for $i = 2, \ldots, r_n$

$$\begin{aligned}\text{var}(Z_{ni}) &= \frac{1}{k}\text{var}\left(\sum_{j=1}^{k} h_i m_{ji}\lambda_j^{\mathrm{T}}u_{(n)i}\right)\\ &= \frac{1}{k}\sum_{j=1}^{k}\text{var}\left(h_i m_{ji}\lambda_j^{\mathrm{T}}u_{(n)i}\right)\\ &= \frac{1}{k}\sum_{j=1}^{k}\lambda_j^{\mathrm{T}}u_{(n)i}u_{(n)i}^{\mathrm{T}}\lambda_j.\end{aligned} \tag{S.43}$$

Results are different for $i = 1$ as the first column of the Hadamard matrix is all ones (P1). For $j, v \in \{1, \ldots, k\}$, $j \neq v$ the covariance is

$$\begin{aligned}\text{cov}\left(h_1 m_{j1}\lambda_j^{\mathrm{T}}u_{(n)1}, h_1 m_{v1}\lambda_v^{\mathrm{T}}u_{(n)1}\right) &= E\left[h_1^2 m_{j1}m_{v1}\lambda_j^{\mathrm{T}}u_{(n)1}\lambda_v^{\mathrm{T}}u_{(n)1}\right]\\ &= E\left[m_{j1}m_{v1}\right]\lambda_j^{\mathrm{T}}u_{(n)1}\lambda_v^{\mathrm{T}}u_{(n)1}\\ &= \lambda_j^{\mathrm{T}}u_{(n)1}\lambda_v^{\mathrm{T}}u_{(n)1}.\end{aligned}$$

From (P1) $m_{j1} = m_{v1} = 1$. Now using the Cauchy-Schwarz inequality,

$$\begin{aligned}\left|\text{cov}\left(h_1 m_{j1}\lambda_j^{\mathrm{T}}u_{(n)1}, h_1 m_{v1}\lambda_v^{\mathrm{T}}u_{(n)1}\right)\right| &= |\lambda_j^{\mathrm{T}}u_{(n)1}||\lambda_v^{\mathrm{T}}u_{(n)1}|\\ &\leq \|\lambda_j\|_2\|u_{(n)1}\|_2\|\lambda_v\|_2\|u_{(n)1}\|_2\\ &\leq \|u_{(n)1}\|_2^2.\end{aligned}$$

The second last last uses the fact that λ is a unit vector and we must have $\|\lambda_j\|_2 \leq 1$, $\|\lambda_v\|_2 \leq 1$ for any j, v. From assumption 1, the right hand side of the previous inequality tends to zero as n tends to infinity. As such $|\text{cov}\left(h_1 m_{j1}\lambda_j^{\mathrm{T}}u_{(n)1}, h_1 m_{v1}\lambda_v^{\mathrm{T}}u_{(n)1}\right)|$ is $o(1)$. Some covariance terms

appear in the expression for var(Z_{n1})

$$\begin{aligned}
\text{var}(Z_{n1}) &= \frac{1}{k}\text{var}\left(\sum_{j=1}^{k} h_1 m_{j1}\lambda_j^{\mathrm{T}} u_{(n)1}\right) \\
&= \frac{1}{k}\sum_{j=1}^{k}\text{var}\left(h_i m_{ji}\lambda_j^{\mathrm{T}} u_{(n)1}\right) + \frac{1}{k}2\sum_{j=1}^{k-1}\sum_{v=j+1}^{k}\text{cov}\left(h_1 m_{j1}\lambda_j^{\mathrm{T}} u_{(n)1}, h_1 m_{v1}\lambda_v^{\mathrm{T}} u_{(n)1}\right) \\
&= \frac{1}{k}\sum_{j=1}^{k}\text{var}\left(h_1 m_{j1}\lambda_j^{\mathrm{T}} u_{(n)1}\right) + \frac{1}{k}2\sum_{j=1}^{k-1}\sum_{v=j+1}^{k}\lambda_j^{\mathrm{T}} u_{(n)1}\lambda_v^{\mathrm{T}} u_{(n)1} \\
&= \frac{1}{k}\sum_{j=1}^{k}\text{var}\left(h_1 m_{j1}\lambda_j^{\mathrm{T}} u_{(n)1}\right) + o(1) \\
&= \frac{1}{k}\sum_{j=1}^{k}\lambda_j^{\mathrm{T}} u_{(n)1} u_{(n)1}^{\mathrm{T}}\lambda_j + o(1) \qquad \text{(S.44)}
\end{aligned}$$

The trailing term can be grouped into an $o(1)$ term as the sketch size k is fixed in our analysis. Using (S.43) and (S.44) we can then determine the row-wise variance totals s_n^2:

$$\begin{aligned}
s_n^2 &= \frac{1}{k}\sum_{i=1}^{r_n}\text{var}(Z_{ni}) \\
&= \frac{1}{k}\sum_{i=1}^{r_n}\sum_{j=1}^{k}\lambda_j^{\mathrm{T}} u_{(n)i} u_{(n)i}^{\mathrm{T}}\lambda_j + o(1) \\
&= \frac{1}{k}\sum_{j=1}^{k}\lambda_j^{\mathrm{T}}\left(\sum_{i=1}^{r_n} u_{(n)i} u_{(n)i}^{\mathrm{T}}\right)\lambda_j + o(1) \\
&= \frac{1}{k}\sum_{j=1}^{k}\lambda_j^{\mathrm{T}} U_{(n)}^{\mathrm{T}} U_{(n)}\lambda_j + o(1) \\
&= \frac{1}{k}\sum_{j=1}^{k}\lambda_j^{\mathrm{T}}\lambda_j + o(1) \\
&= \frac{1}{k} + o(1).
\end{aligned}$$

The step in the last line follows as we have taken λ to be a unit vector. The fact that $U_{(n)}^{\mathrm{T}} U_{(n)} = I_d$ for all n serves as a useful normalisation to give stable limiting behaviour. We are working with a triangular array where the rows are nearly standardised. Asymptotically in n, $s_n^2 \to 1/k$.

We now establish a sequence of upper bounds (K_n). As the random variables in the construction of construction of the Hadamard sketch are bounded, we can bound the random variables in the triangular array (S.40) using the leverage scores of the sequence of source datasets. Now as the random sign $h_i \in \{+1, -1\}$ we have that for all $i = 1, \dots, r_n$:

$$|Z_{ni}| = \frac{1}{\sqrt{k}}|h_i \sum_{j=1}^{k} m_{ji}\lambda_j^{\mathrm{T}} u_{(n)i}| = \frac{1}{\sqrt{k}}|\sum_{j=1}^{k} m_{ji}\lambda_j^{\mathrm{T}} u_{(n)i}|.$$

Now using the Cauchy-Schwarz inequality,

$$\frac{1}{\sqrt{k}}|\left(\sum_{j=1}^{k} m_{ji}\lambda_j^{\mathrm{T}}\right) u_{(n)i}| \leq \frac{1}{\sqrt{k}}\|\left(\sum_{j=1}^{k} m_{ji}\lambda_j\right)\|_2\|u_{(n)i}\|_2. \tag{S.45}$$

Using the triangle inequality,

$$\|\left(\sum_{j=1}^{k} m_{ji}\lambda_j\right)\|_2 \leq \sum_{j=1}^{k}\|m_{ji}\lambda_j\|_2. \tag{S.46}$$

Now as $m_{ji} \in \{+1, -1\}$ for all $j = 1, \ldots, k$,

$$\sum_{j=1}^{k}\|m_{ji}\lambda_j\|_2 = \sum_{j=1}^{k}\|\lambda_j\|_2. \tag{S.47}$$

As λ is a unit vector we can easily form the bound

$$\sum_{j=1}^{k}\|\lambda_j\|_2 \leq k. \tag{S.48}$$

Substituting (S.47) and (S.48) into (S.45) leads to the upper bound for $i = 1, \ldots, r_n$:

$$|Z_{ni}| \leq \sqrt{k}\|u_{(n)i}\|_2. \tag{S.49}$$

We can then form the sequence of upper bounds K_n:

$$K_n = \sqrt{k}\max_{i=1,\ldots,r_n}\|u_{(n)i}\|_2.$$

We have that $|Z_{ni}| \leq K_n$ almost surely for $i = 1, \ldots, r_n$ and $n \in \mathbb{N}$. As the sketch size k is fixed in our analysis, and using Assumption 1:

$$\lim_{n\to\infty} K_n = \sqrt{k}\lim_{n\to\infty}\max_{i=1,\ldots,r_n}\|u_{(n)i}\|_2 = 0.$$

As $s_n^2 = 1/k + o(1)$ we have an asymptotically standardised array, thus in the limit $K_n/s_n \to 0$. We can use Theorem S.2 to conclude that the triangular array of random variables defined in (S.40) satisfies Lindeberg's condition. As such, the conditions of Theorem S.1 are satisfied. We conclude that the row sums in (S.41) converge in distribution to $N(0, 1/k)$. Finally, the Cramér-Wold device gives that the whitened sketched dataset has a limiting matrix normal distribution. That is the sequence of random matrices $\widetilde{A}V_{(n)}D_{(n)}^{-1}$ converges in distribution to a $MN\left(0, I_k, I_d/k\right)$ random matrix.

6.3. *Simulation*

We assessed the finite sample behaviour of the central limit approximation at different levels of k and p. We dropped some predictors from the full flights dataset discussed in Section 6.2 of the main text to give smaller datasets with $p = 10$ and $p = 25$ covariates. We then took subsamples of different sizes from each of the datasets. A single subsample was taken at each value of n, so the same subsampled dataset was being sketched each time. One thousand sketches were taken of each dataset at different values of k. We tested the joint multivariate normality of $(\widetilde{y}, \widetilde{X})$ and the normality of the sketched residual $\widetilde{e} = S(y - X\beta_F)$. The squared Mahalanobis distance of the sketched observations was compared to the theoretical χ^2-distribution. As n increases the

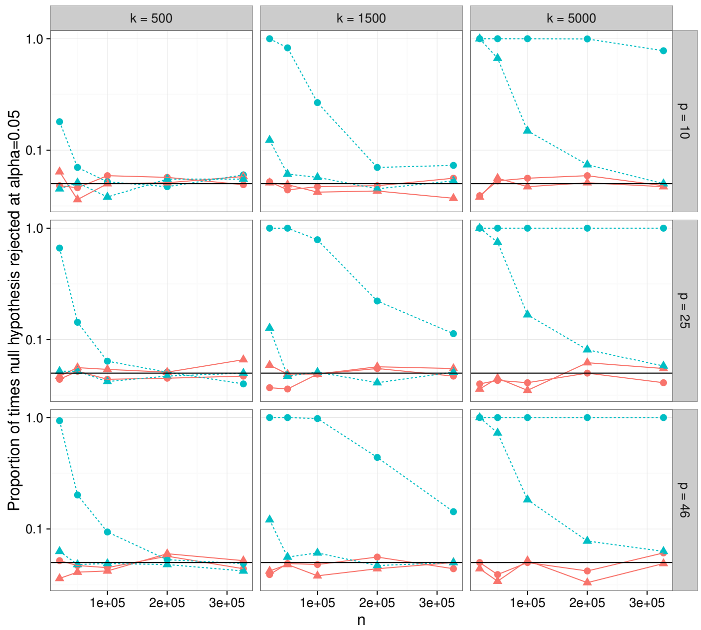

Fig. 3. Proportion of times null hypothesis of normality is rejected against size of the source dataset (n) for the Hadamard (solid line) and Clarkson-Woodruff sketches (dashed line). Results for tests of the sketched residual vector $\widetilde{e} = S(y - X\beta_F)$ are plotted as triangles $(\triangle)$, and results for tests of the entire sketched dataset $(\widetilde{y}, \widetilde{X})$ are plotted as circles $(\circ)$. The horizontal line gives the type 1 error of 0.05. The y-axis is on a log scale.

rejection rate is expected to fall to the type one error rate of 0.05. Figure 3 plots the proportion of times the null hypothesis of normality is rejected against the size of the source dataset.

The Hadamard sketch appears to have a much faster rate of convergence than the Clarkson-Woodruff sketch. When using a Hadamard sketch, each row in the sketched dataset is a linear combination of n observations from the source dataset. When using a Clarkson-Woodruff sketch, each row in the sketched dataset is expected to be a combination of only n/k observations from the source dataset. As such, n/k must be large for the normal approximation to hold. As expected, the rejection rate for the Clarkson-Woodruff sketch increases with k, but remains stable for the Hadamard sketch. The Hadamard sketch seems to be less sensitive to the number of covariates. The extra $\log k$ computation cost associated with the Hadamard sketch appears to have the benefit of accelerated convergence to normality. As $\widetilde{y} = \widetilde{X}\beta_F + \widetilde{e}$, normality of the sketched residual is perhaps sufficient in justifying the approximate confidence intervals using Theorem 1 (ii). The sketched residual converges much more quickly than the full sketched data matrix, which perhaps explains the good coverage properties of the confidence intervals for β_S in Table 2.

7. PROOF OF THEOREM 3

Notation is slightly heavier in the proof compared to the main text for the sake of clarity. Again we do not explicitly condition on the source dataset $A_{(n)}$, the source dataset is always fixed, and the only randomness is from the sketching matrix. The sketched data will be denoted $\widetilde{y}_{(n)}$ and

$\widetilde{X}_{(n)}$ to denote the dependence on the $n \times d$ source dataset. So $\widetilde{y}_{(n)} = Sy_{(n)}$ and $\widetilde{X}_{(n)} = SX_{(n)}$. The dimension of the sketched dataset does not change.

Assumption 2 is of assistance in establishing the limit theorem. Let

$$Q_{(n)} = n^{-1} \begin{pmatrix} y_{(n)}^{\mathrm{T}} y_{(n)} & y_{(n)}^{\mathrm{T}} X_{(n)} \\ X_{(n)}^{\mathrm{T}} y_{(n)} & X_{(n)}^{\mathrm{T}} X_{(n)} \end{pmatrix}.$$

The matrix $Q_{(n)}$ contains the sufficient statistics needed to fit a Gaussian linear model, $y_{(n)}^{\mathrm{T}} y_{(n)}, X_{(n)}^{\mathrm{T}} y_{(n)}$ and $X_{(n)}^{\mathrm{T}} X_{(n)}$ given the source dataset $A_{(n)} = (y_{(n)}, X_{(n)})$. Assumption 2 states the averaged sufficient statistic matrix converges to a limiting matrix Q. It will be helpful to partition the limiting matrix Q as

$$Q = \lim_{n\to\infty} n^{-1} \begin{pmatrix} y_{(n)}^{\mathrm{T}} y_{(n)} & y_{(n)}^{\mathrm{T}} X_{(n)} \\ X_{(n)}^{\mathrm{T}} y_{(n)} & X_{(n)}^{\mathrm{T}} X_{(n)} \end{pmatrix} = \begin{pmatrix} s & m^{\mathrm{T}} \\ m & G \end{pmatrix}, \tag{S.50}$$

where s is a scalar, G is a $p \times p$ matrix and b is a p-length column vector. The matrix G is the limiting averaged Gram matrix of the predictors. The vector m is the limit of the predictor response inner products $n^{-1} X_{(n)}^{\mathrm{T}} y_{(n)}$, and the scalar s is the limit of the mean total sum of squares $n^{-1} y_{(n)}^{\mathrm{T}} y_{(n)}$.

As mentioned, the assumption of a sequence of source datasets also gives a sequence of optimal least squares coefficients and residual errors. Let $\sigma_F^{2(n)} = RSS_F/n$. Define the limiting least squares coefficient estimate as $\beta = \lim_{n\to\infty} \beta_F^{(n)}$ and the limiting residual error as $\sigma^2 = \lim_{n\to\infty} \sigma_F^{2(n)}$. Both β and σ^2 can be expressed as functions of the matrix Q. Specifically,

$$\beta = G^{-1} m, \tag{S.51}$$

$$\sigma^2 = s - m^{\mathrm{T}} G^{-1} m. \tag{S.52}$$

From Assumption 2, we have that $n^{-1} V_{(n)} D_{(n)}^2 V_{(n)}^{\mathrm{T}} \to Q$. As such we have that $n^{-1/2} D_{(n)} V_{(n)}^{\mathrm{T}} \to Q^{1/2}$ From the sketching central limit theorem the whitened sketched data converges to a matrix normal distribution

$$(\widetilde{y}_{(n)}, \widetilde{X}_{(n)}) V_{(n)} D_{(n)}^{-1} \xrightarrow{d} \mathrm{MN}\left(0, I_k, I_d/k\right)$$

The benefit of adding Assumption 2 is that using Slutsky's theorem we have the additional convergence result

$$n^{-1/2} (\widetilde{y}_{(n)}, \widetilde{X}_{(n)}) \xrightarrow{d} \mathrm{MN}\left(0, I_k, Q/k\right).$$

To prove results (i) and (ii) we use the continuous mapping theorem (Van Der Vaart, 1998, p. 7) in conjunction with the previous convergence result. It will be helpful to define the random variables $\widetilde{y}_L, \widetilde{X}_L$ as having the above limiting matrix normal distribution

$$(\widetilde{y}_L, \widetilde{X}_L) \sim MN\left(0, I_k, Q/k\right).$$

This is so we can say that

$$n^{-1/2} (\widetilde{y}_{(n)}, \widetilde{X}_{(n)}) \xrightarrow{d} (\widetilde{y}_L, \widetilde{X}_L).$$

As $\widetilde{X}_L$ is of rank p almost surely, and $\widetilde{X}_{(n)} \xrightarrow{d} \widetilde{X}_L$ we can apply the continuous mapping theorem to determine the limiting distribution of β_S. The random matrix $(\widetilde{y}_L, \widetilde{X}_L)$ can be described using

a hierarchical model completely analogous in structure to the hierarchical model established for the Gaussian sketch in Section 3.1 of the main text. Specifically,

$$\widetilde{y}_L \mid \widetilde{X}_L \sim N\left(\widetilde{X}_L\beta, \frac{1}{k}\sigma^2 I_k\right),$$
$$\widetilde{X}_L \sim \mathrm{MN}\left(0, I_k, \frac{1}{k}G\right).$$

From Theorem 1 in the main text, and defining the function g to output β_S, we have that

$$g(\widetilde{y}_L, \widetilde{X}_L) \sim \text{Student}\left(\beta,\ \frac{\sigma^2}{k-p+1}G^{-1},\ k-p+1\right).$$

As such, for the Hadamard and Clarkson-Woodruff sketches,

$$\{\beta_S \mid y_{(n)}, X_{(n)}\} \xrightarrow{d} \text{Student}\left(\beta,\ \frac{\sigma^2}{k-p+1}G^{-1},\ k-p+1\right).$$

Let

$$C_{(n)} = \sigma_F^{2(n)}/(k-p+1)\left(n^{-1}X_{(n)}^{\mathrm{T}}X_{(n)}\right)^{-1}.$$

Now as $n^{-1}X_{(n)}^{\mathrm{T}}X_{(n)} \to G$, $\sigma_F^{2(n)} \to \sigma^2$, and $\beta_F^{(n)} \to \beta$, Slutsky's theorem can be used to arrive at (i),

$$C_{(n)}^{-1/2}(\beta_S - \beta_F) \xrightarrow{d} \text{Student}\left(0,\ I_p,\ k-p+1\right).$$

For result (ii), let us define the function

$$\begin{aligned} f(n^{-1/2}\widetilde{y}_{(n)}, n^{-1/2}\widetilde{X}_{(n)}) &= \left\{\left(n^{-1}\widetilde{X}_{(n)}^{\mathrm{T}}\widetilde{X}_{(n)}\right)^{+}\right\}^{-1/2}\left(\widetilde{X}_{(n)}^{+}\widetilde{y}_{(n)} - \beta\right) \\ &= \left\{n\left(\widetilde{X}_{(n)}^{\mathrm{T}}\widetilde{X}_{(n)}\right)^{+}\right\}^{-1/2}(\beta_S - \beta). \end{aligned}$$

This function transforms β_S so that the output vector is uncorrelated. This function is also continuous over the set where $\widetilde{X}_{(n)}$ is of rank p. Again using the fact that $\widetilde{X}_L$ has rank p almost surely, it follows from the continuous mapping theorem that $f(n^{-1/2}\widetilde{y}_{(n)}, n^{-1/2}\widetilde{X}_{(n)}) \xrightarrow{d} f(\widetilde{y}_L, \widetilde{X}_L)$. Result (ii) in Theorem 1 also applies to the hierarchical model for $\widetilde{y}_L, \widetilde{X}_L$, and gives the distribution of the transformed β_S under the Gaussian sketch. The distribution of $f(\widetilde{y}_L, \widetilde{X}_L)$ will be

$$f(\widetilde{y}_L, \widetilde{X}_L) \sim N\left(0, \frac{\sigma^2}{k}I_p\right).$$

As such, for the Clarkson-Woodruff and Hadamard sketches,

$$\left\{n\left(\widetilde{X}_{(n)}^{\mathrm{T}}\widetilde{X}_{(n)}\right)^{+}\right\}^{-1/2}(\beta_S - \beta) \xrightarrow{d} N\left(0, \frac{\sigma^2}{k}I_p\right).$$

Now let

$$\widetilde{C}_{(n)} = n\sigma_F^{2(n)}/k\left(\widetilde{X}_{(n)}^{\mathrm{T}}\widetilde{X}_{(n)}\right)^{+}.$$

As $\sigma_F^{2(n)} \to \sigma^2$, and $\beta_F^{(n)} \to \beta$, Slutsky's theorem can be used to arrive at (ii)

$$\widetilde{C}_{(n)}^{-1/2}(\beta_S - \beta_F^{(n)}) \xrightarrow{d} N(0, I_p).$$

8. Proof of Theorem 4

Application of the continuous mapping theorem gives that the distribution of β_S and β_P^* under the Hadamard and Clarkson-Woodruff sketches converges to the distribution of the estimators under the Gaussian sketch. This does not necessarily guarantee convergence in moments. To establish a limit theorem for the bias and variance of the estimators, we need a uniform integrability condition on the sketched dataset. The sketched data will be denoted $\widetilde{X}_{(n)}$ to denote the dependence on the $n \times p$ source covariate matrix. So $\widetilde{X}_{(n)} = SX_{(n)}$.

Let $G_{(n)} = n^{-1}\widetilde{X}_{(n)}^{\mathrm{T}}\widetilde{X}_{(n)}$. From the continuous mapping theorem and Theorem 2 in the main text, it is known that $G_{(n)}^{-1} \mid A_{(n)}$ converges in distribution to W where W has an Inverse-Wishart(k, kQ^{-1}) distribution and Q is the limiting matrix from Assumption 2. We would like to establish convergence in first and second moments, that is $E(G_{(n)}^{-1} \mid A_{(n)}) \to E(W)$, var$(G_{(n)}^{-1} \mid A_{(n)}) \to$ var(W). If convergence in first and second moments occurs, then we can show that (i) and (ii) will hold. If $E(G_{(n)}^{-1} \mid A_{(n)}) \to E(W)$, we can say that $E(\beta_P^* - \beta \mid A_{(n)}) \to 0$ where β is the limiting ordinary least squares estimator (S.51), that is a function of the limiting matrix Q in Assumption 2. From here, using that $\lim_{n\to\infty} \beta_F^{(n)}$, Slutsky's theorem can be used to arrive at (i)

$$E(\beta_P^* - \beta_F^{(n)} \mid A_{(n)}) \to 0.$$

To show convergence of the variance of the sketched estimator (ii), we define

$$C_{(n)} = \frac{(k-p-1)}{(k-p)(k-p-3)} \left\{ MSS_F^{(n)} \left(X_{(n)}^{\mathrm{T}} X_{(n)} \right)^{-1} + \frac{(k-p+1)}{(k-p-1)} \beta_F^{(n)} \beta_F^{(n)\mathrm{T}} \right\},$$
$$C = \frac{(k-p-1)}{(k-p)(k-p-3)} \left\{ (s-\sigma^2) G^{-1} + \frac{(k-p+1)}{(k-p-1)} \beta\beta^{\mathrm{T}} \right\}.$$

Where s, σ^2 and and G are functions of the limiting matrix Q, as in (S.50), (S.51) and (S.52). If var$(G_{(n)}^{-1} \mid A_{(n)}) \to$ var(W) it follows that

$$\mathrm{var}_S \left(C^{-1/2}(\beta_P^* - \beta) \mid A_{(n)} \right) \to I_p.$$

As $C_{(n)}$ converges to C and $\beta_F^{(n)}$ converges to β asymptotically with n, an application of Slutsky's theorem gives (ii),

$$\mathrm{var}_S \left(C_{(n)}^{-1/2}(\beta_P^* - \beta_F^{(n)}) \mid A_{(n)} \right) \to I_p.$$

As such, if we can establish that var$(G_{(n)}^{-1} \mid A_{(n)}) \to$ var(W) we have proved (ii). The following theorem describes the necessary conditions for such convergence to occur.

Theorem S.3. *(Billingsley, 1968, Theorem 5.4) Let $X_1, \ldots, X_n$ be a sequence of random vectors. Suppose X_n converges in distribution to a random variable Z as n tends to infinity. For the additional convergence of moments $E[X_n] \to E[Z]$ and var$[X_n] \to$ var$[Z]$, it must hold that*

for all conformable constant vectors λ

$$\lim_{M\to\infty} \limsup_{n\to\infty} |\lambda^{\mathrm{T}} X_n|^2 \mathbb{1}_{\{|\lambda^{\mathrm{T}} X_n|^2 \geq M\}} = 0.$$

The above condition can be difficult to verify directly. It can be shown that if asymptotically $|\lambda^{\mathrm{T}} X_n|$ has a bounded fourth moment, then the integrability condition is satisfied (Van Der Vaart, 1998, section 2.5).

A linear combination of the elements of the random matrix $G_{(n)}^{-1}$ can be written an $\text{trace}(\Lambda G_{(n)}^{-1})$ for a $p \times p$ matrix of constants Λ. From theorem S.3, it is sufficient to show that show that the expected value of $|\text{trace}(\Lambda G_{(n)}^{-1})|^4$ is finite for large n to show the desired convergence in moments.

As $\text{trace}(\Lambda G_{(n)}^{-1})$ equals the sum of the singular values of the matrix $\Lambda G_{(n)}^{-1}$, we can form an upper bound on the value,

$$\text{trace}(\Lambda G_{(n)}^{-1}) \leq p \|\Lambda G_{(n)}^{-1}\|_2 \leq p \|\Lambda\|_2 \|G_{(n)}^{-1}\|_2.$$

Squaring both sides gives an upper bound on the quantity that must satisfy the uniform integrability condition,

$$|\text{trace}(\Lambda G_{(n)}^{-1})|^2 \leq p^2 \|\Lambda\|_2^2 \|G_{(n)}^{-1}\|_2^2.$$

Squaring again gives an upper bound on the fourth moment of the linear combination of interest

$$\begin{aligned} |\text{trace}(\Lambda G_{(n)}^{-1})|^4 &\leq p^4 \|\Lambda\|_2^4 \|G_{(n)}^{-1}\|_2^4 \\ &= p^4 \|\Lambda\|_2^4 \{1/\sigma_{\text{min}}^4(G_{(n)})\}. \end{aligned}$$

By Assumption 3, the expectation of the right hand side is finite. As such, the uniform integrability condition holds and we can conclude that $E(G_{(n)}^{-1} \mid A_{(n)}) \to E(W), \text{var}(G_{(n)}^{-1} \mid A_{(n)}) \to \text{var}(W)$. As discussed at the beginning of the proof this is sufficient to show that (i) and (ii) hold.

9. UNCONDITIONAL RESULTS

The unconditional variance of the Gaussian sketch can be obtained using the law of total variance, Theorem 1 (i) in the main text, and properties of the ordinary least squares estimator $E_y(\beta_F \mid X) = \beta_0, \text{var}_y(\beta_F \mid X) = \sigma^2(X^{\mathrm{T}}X)^{-1}, E_y(RSS_F \mid X) = (n-p)\sigma^2, E_y(MSS_F \mid X) = p\sigma^2 + n\gamma^2$. Recall that $E_S(\beta_S \mid y, X) = \beta_F$ and $E_S(\beta_P^* \mid y, X) = \beta_F$. For the complete sketching estimator

$$\begin{aligned} \text{var}_y(\beta_S \mid X) &= E_y\{\text{var}_S(\beta_S \mid y, X)\} + \text{var}_y\{E_S(\beta_S \mid y, X)\} \\ &= E_y\left\{\frac{RSS_F}{(k-p-1)}(X^{\mathrm{T}}X)^{-1} \mid X\right\} + \sigma^2(X^{\mathrm{T}}X)^{-1} \\ &= \frac{(n-p)\sigma^2}{(k-p-1)}(X^{\mathrm{T}}X)^{-1} + \sigma^2(X^{\mathrm{T}}X)^{-1}. \end{aligned} \tag{S.53}$$

We can also determine the unconditional properties of the partial sketch estimator β_P^* using equation (4) in the main text. Using the law of total variance

$$
\begin{aligned}
\mathrm{var}_y(\beta_P^* \mid X) &= E_y\left\{\mathrm{var}_S(\beta_P^* \mid y, X)\right\} + \mathrm{var}_y\left\{E_S(\beta_P^* \mid y, X)\right\} \\
&= E_y\left[\frac{(k-p-1)}{(k-p)(k-p-3)}\left\{MSS_F(X^{\mathrm{T}}X)^{-1} + \frac{k-p+1}{k-p-1}\beta_F\beta_F^{\mathrm{T}}\right\} \mid X\right] + \sigma^2(X^{\mathrm{T}}X)^{-1} \\
&= \frac{(k-p-1)}{(k-p)(k-p-3)}\left[(p\sigma^2 + n\gamma^2)(X^{\mathrm{T}}X)^{-1} + \frac{k-p+1}{k-p-1}\{\sigma^2(X^{\mathrm{T}}X)^{-1} + \beta_0\beta_0^{\mathrm{T}}\}\right] + \\
&\quad \sigma^2(X^{\mathrm{T}}X)^{-1}. \qquad \text{(S.54)}
\end{aligned}
$$

REFERENCES

ANDERSON, I. (1997). *Combinatorial Designs and Tournaments*. Oxford lecture series in mathematics and its applications. Clarendon Press.

BILLINGSLEY, P. (1968). *Convergence of Probability Measures*. New York: Wiley.

BILLINGSLEY, P. (1999). *Convergence of Probability Measures*. New York: Wiley.

GELMAN, A., CARLIN, J. B., STERN, H. S., DUNSON, D. B., VEHTARI, A. & RUBIN, D. B. (2014). *Bayesian Data Analysis*. Boca Raton: Chapman & Hall, 3rd ed.

LETAC, G. & MASSAM, H. (2004). All invariant moments of the Wishart distribution. *Scandinavian Journal of Statistics* **31**, 295–318.

PRUSS, A. R. & SZYNAL, D. (2000). On the central limit theorem for negatively correlated random variables with negatively correlated squares. *Stochastic Processes and their Applications* **87**, 299 – 309.

SEARLE, S. R. (1997). *Linear Models*. New Jersey: Wiley-Interscience.

SVANTE, J. (1988). Some pairwise independent sequences for which the central limit theorem fails. *Stochastics* **23**, 439–448.

VAN DER VAART, A. (1998). *Asymptotic Statistics*. Cambridge Series in Statistical and Probabilistic Mathematics, 3. Cambridge University Press.

[*Received on* 2 *January* 2017. *Editorial decision on* 1 *April* 2017]
