# Supplementary material for: Statistical properties of sketching algorithms
Source: Biometrika. Author manuscript; Available in PMC 2022 Feb 4. (PMC7612324; doi:10.1093/biomet/asaa062)
Supplement: Supplementary Material [file EMS140909-supplement-Supplementary_Material.zip › sketching_biometrika.zip!/styleguide-miscellanea.pdf]

Biometrika (2018), **103**, 1, *pp*. 1–8
Printed in Great Britain Advance Access publication on 31 July 2018

Some notes on *Biometrika* style

BY A. C. DAVISON
Institute of Mathematics, Ecole Polytechnique Fédérale de Lausanne, Station 8, 1015 Lausanne, Switzerland
anthony.davison@epfl.ch

P. FEARNHEAD
Department of Mathematics and Statistics, Lancaster University, Bailrigg, Lancaster LA1 4YF, U.K.
editor@biometrika.org.uk

R. GESSNER
Department of Statistical Science, University of London, Gower Street, London WC1E 6BT, U.K.
editorial@biometrika.org.uk

AND D. M. TITTERINGTON
Department of Statistics, University of Glasgow, Glasgow G12 8QQ, U.K.
mike@stats.gla.ac.uk

SUMMARY

There should be a single paragraph summary which should not contain formulae or symbols, followed by some key words in alphabetical order. Typically there are 3–8 key words, which should contain nouns and be singular rather than plural. The summary contains bibliographic references only if they are essential. It should indicate results rather than describe the contents of the paper: for example, 'A simulation study is performed' should be replaced by a more informative phrase such as 'In a simulation our estimator had smaller mean square error than its main competitors.'

Some key words: Address; Appendix; Figure; Length; Reference; Style; Summary; Table.

1. INTRODUCTION

These notes are intended both as a guide to journal style and as an example file for authors intending to prepare a paper using the *Biometrika* LaTeX class.

Before preparing a paper for submission, please examine a recent issue carefully and follow that arrangement of sections, formulae, references, tables, etc. closely. In particular please note the points given below. The preparation for publication of a paper that is technically acceptable, but which does not follow these requirements, would involve the editors in a large amount of work, so it may be necessary to return such a manuscript for reformatting, leading to a delay in publication.

Authors should not attempt to control page spacing in LaTeX. Details such as the large white spaces around this section are corrected by the production team.

Biometrika papers do not contain a 'contents of the paper' paragraph.

© 2018 Biometrika Trust

2. ADDRESS

For each author please give one postal address, including a department, postcode and country, and one e-mail address; these should be the best permanent addresses current at time of publication. Acknowledgements to other institutions should be put with other acknowledgements at the end of the paper. Names of states should be given in full, thus: California rather than CA, São Paulo rather than SP. Use U.S.A. and U.K. Note that England, Scotland and Wales should not be used.

3. LENGTH

The average length for papers published in recent years is just under 13 sides. The probability of acceptance drops sharply beyond this length, particularly if it is felt that a paper is long in relation to its original content. Authors should endeavour to write as concisely as possible, consistent with clarity. Long or standard derivations should be omitted, referenced elsewhere, or made available in a supplementary document on the *Biometrika* web site; see page 7. Essential technical details may be placed in an appendix.

The maximum length for a paper in the Miscellanea section is 8 journal sides.

4. STYLE

4.1. *Sections, subsections and paragraphs*

If subsections are used to divide a section, no text should appear before the first subsection; all text should appear within numbered subsections. Subsubsections are not used.

The end of a paragraph is marked in the .tex file by a blank line. Extra characters such as \\ at the end of lines or paragraphs should not be used. Bad line breaks are corrected during the production process.

4.2. *Spelling, abbreviations and special symbols*

English spelling is used, with Oxford "-ize" endings.

Verbal phrases inside brackets or dashes, or in italic or bold type, should not be used. Quotation marks should be used only for direct quotations, which should be attributed. Footnotes should be avoided except for tables.

Abbreviations like a.s., i.i.d., d.f., w.r.t., ANOVA, AR, MCMC, MAP and ML may not be used. Exceptions to this are common non-mathematical abbreviations such as DNA and HIV, which appear as ordinary upper-case letters.

Do not create abbreviations to describe methods. Thus 'our method is more efficient than Wellner and Zhang's method' should replace 'our new method is better than method WZ'.

Special symbols like $\xrightarrow{d}$, $\forall$, $\exists$, $:=$ and $=:$ should not be used. The symbol $|$ should not be used in text as shorthand, and in mathematics the TeX symbol `\mid` should be used to denote conditioning, rather than $|$ or `\vert`.

Symbols comprising several letters such as AIC or AR(p) may be used as mathematical objects if previously defined. They may not be used as abbreviations for English words; thus 'the AR model' should be 'the autoregressive model'. In such cases small capital letters, for example the TeX syntax `\textsc{aic}` for AIC, are used; consistency is best assured by defining a macro at the start of the `.tex` file.

One of the most common reasons that publication of scientifically acceptable papers is delayed is authorial failure to adhere to journal policy on abbreviations, so it may be worthwhile to explain why *Biometrika* eschews them. The purpose of scientific writing is to convey ideas as clearly and directly as possible. Abbreviations militate against this: a reader who does not know them will spend time looking back through the paper to find what they mean, and they lead to sloppy mechanistic writing. A sentence such as 'MLE for a GLMM may be performed using the BFGS, NR, CG or EM algorithms, but MCMC is an alternative' forces the reader to waste energy on parsing acronyms rather than focusing on the underlying ideas.

4.3. *English*

English sentences containing mathematical expressions or displayed formulae should be punctuated in the usual way: in particular please check carefully that all displayed expressions are correctly punctuated. Displayed expressions should be preceded by a colon only if grammatically warranted. Do not place a colon in the middle of a clause.

Words in common terms such as central limit theorem or Brownian motion are capitalized only if they are derived from proper names: thus bootstrap, lasso and mean square error rather than Bootstrap, Lasso and Mean Square Error.

Hyphens - (- in TEX), n-dashes – (--), m-dashes — (---), and minus signs − ($-$) have different uses. Hyphens are used to join two words, or in the double-barrelled name of a single person (e.g. non-user, Barndorff-Nielsen); n-dashes are used in ranges of numbers or to join the names of two different people (1–7, Neyman–Pearson); and minus signs are used in mathematics (e.g. -2). m-dashes are not used in *Biometrika*. Parenthetical remarks, like this subordinate clause, are placed between two commas.

Two bugbears: the phrase ‘note that’ can almost always be deleted, and the phrase ‘is given by’ should be cut to ‘is’ in a sentence such as ‘The average is given by $\bar{X} = n^{-1}(X_1 + \cdots + X_n)$.

4.4. *Mathematics*

Equation numbers should be included only when equations are referred to; the numbers must be placed on the right. Long or important mathematical, not verbal, expressions should be displayed, i.e., shown on a separate line. Short formulae should be left in the text to save space where possible, but must not be more than one line high and not contain reduced-size type. For example $\frac{\mathrm{d}y}{\mathrm{d}x}$ must not be left in the text, but should be written $\mathrm{d}y/\mathrm{d}x$ or it should be displayed. Likewise write $n^{1/2}$ not $n^{\frac{1}{2}}$. Also $\begin{pmatrix} a \\ b \end{pmatrix}$ and suchlike expressions must not be left in the text. Equations involving lengthy expressions should, where possible, be avoided by introducing suitable notation.

Symbols should not start sentences. Distinctive type, e.g., boldface, for matrices and vectors is not used in *Biometrika*. Vectors are assumed to be column vectors, unless explicitly transposed. The use of an apostrophe to denote matrix or vector transposition should be avoided; it is preferable to write A^{T}, a^{T}. Capital script letters may be used sparingly, typically to denote sets, but care should be taken as some are hard to distinguish.

Please arrange brackets in the order $[\{(\,)\}]$, iterating as necessary, and follow the usual conventions for e, exp, use of solidus, square root signs and so forth as in a recent issue. The sign $\sqrt{}$ is not used, and the sign $\surd$ is used only sparingly; powers of complicated quantities should be represented as $(mnpq)^a$.

Multiple overbars such as $\bar{\bar{x}}$ must be avoided, as must $\widehat{ab}$, $\widehat{(a+b)}$, $\widehat{\mathrm{var}}$, $\overline{ab}$, $\overline{(a+b)}$ and symbols with underbars. Subscripts and superscripts, and second-order sub- and superscripts, should be aligned horizontally. Avoid sub- and superscripts of third, and greater, order.

Please use: var(x) not var x or Var(x); cov not Cov; pr for probability not Pr or P; tr not trace; $E(X)$ for expectation not EX or $\mathcal{E}(X)$; $\log x$ not $\log_e x$ or $\ln x$; rth not r-th or r^{th}. Please avoid: ‘$\cdot$’ or ‘.’ for product; a/bc, which should be written $a/(bc)$ or $a(bc)^{-1}$. Use the form $x_1, \ldots, x_n$ not $x_1, x_2, \ldots x_n$ and $\sum_{i=1}^{n}$ not $\sum_{1}^{n}$. Zeros precede decimal points: 0.2 not .2.

The use of ‘$\cdots$’ and ‘$\ldots$’ is $\ldots$ in lists, such as $y_1, \ldots, y_n$, and $\cdots$ between binary operators, giving $y_1 + \cdots + y_n$. Ranges of integers are denoted $i = 1, \ldots, n$, whereas $0 \leq x \leq 1$ is used for ranges of real numbers.

Biometrika deprecates the appearance of words in displayed equations, which should be formatted as

$$\bar{Y} = n^{-1} \sum_{j=1}^{n} Y_j, \quad S^2 = \sum_{j=1}^{n} (Y_j - \bar{Y})^2; \qquad (1)$$

note the punctuation and space between the expressions. Displays such as (1) should take no more space than necessary, being placed on a single line where possible. Displayed mathematical expressions should

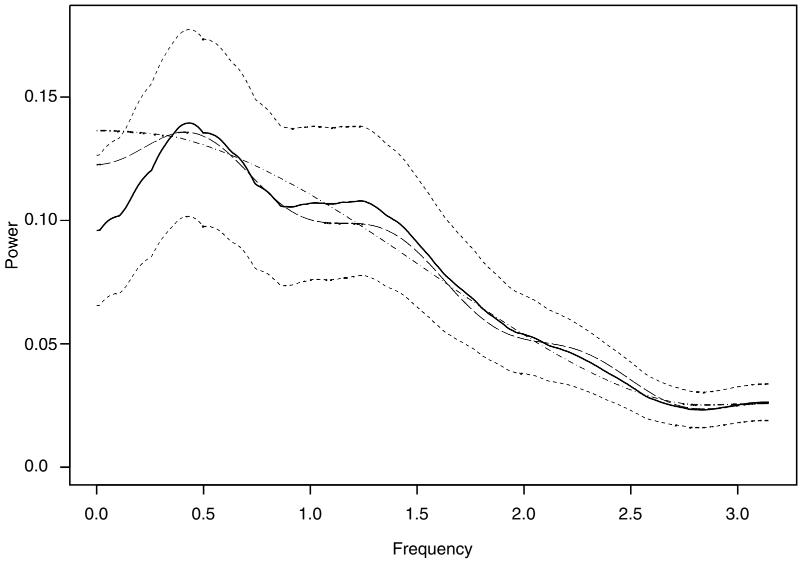

Fig. 1. A graph showing the truth (dot-dash), an estimate (dashes), another estimate (solid), and 95% pointwise confidence limits (small dashes).

be punctuated thus: indexed equations and similar quantities in text are formatted as $y_j = x_j^{\mathrm{T}}\beta + \varepsilon_j$ $(j = 1, \ldots, n)$, and are displayed as

$$y_{ij} = x_j^{\mathrm{T}}\beta_i + \varepsilon_{ij} \quad (i = 1, \ldots, m;\ j = 1, \ldots, n).$$

References to sequences of equations are (1)–(3), not (1–3).

4.5. *Figures*

Figures are a common source of delay during production, usually because elementary guidelines have not been respected. General comments may be found in the document 'RSSGraphs.pdf' enclosed with the *Biometrika* formatting files, and more detail is given in standard references such as Cleveland (1993, 1994) or Tufte (1983).

All the elements of a graph, including axis labels, should be large enough to be read easily, so the graph should be given a shape that will use the page space well. The use of large symbols, such as $\times$, for points should be avoided. If both axes of a panel show the same quantities, the panel should usually be square. Many graphs are made using the statistical environment `R` (R Development Core Team, 2018). If so, they should be made at roughly the size at which they will appear in the journal. Usually graphs reduced from A4 or US page sizes must be remade to ensure their legibility.

Check that all the axes are labelled correctly and include units of measurement. Axis labels should have the format 'Difference of loglikelihoods': only the initial letter of the first word is upper-case. The numbers on the vertical axis should be parallel to the horizontal axis, and should be in the same font as the text; normally the change of font is left to the production process, but it is helpful if the numbers are placed horizontally.

A panel should not contain an inset defining the line-types and symbols; this description should appear in the caption.

Please submit figures in greyscale wherever possible. *Biometrika* publishes in colour where this is essential, but care should be taken to ensure that any colours chosen will be distinguishable on the cream paper used for the journal, i.e., yellow should be avoided.

Figures should be referred to consecutively by number. Use of the LaTeX `\label` and `\ref` commands to refer to figures and tables helps to reduce errors and so is preferred. Figure 1 is a reference to a figure at the start of a sentence, whereas subsequent references are abbreviated, for example to Fig. 1.

Table 1. *Perceptions about racial groups in the U.S. population*

	2000 Census percent of U.S. population	Mean percent estimated for U.S. population		
		White Rs	Black Rs	Hispanic Rs
White	75	59	56	60
Black	12	30	38	40
Asian	4	16	21	30
American Indian	1	13	17	23
More than two races	2	41	48	50
Hispanic	13	23	27	42

U.S., United States of America; R, respondent.

4.6. *Tables*

Tables should be referred to consecutively by number. Table is not abbreviated to Tab.

Check that the arrangement makes effective use of the *Biometrika* page. Layouts that have to be printed sideways should be avoided if possible. For this reason tables should not be more than 92 characters wide, including decimal points and brackets (1 character), and minus and other signs and spaces (at least 2 characters). Rules are not used in *Biometrika* tables, which should be arranged to be clear without them.

Often tables can be improved by multiplying all the entries by a power of ten, so that 0.002 and 0.02 become 2 and 20 respectively, for example; this will often both save space and convey the message of the table more effectively. Table 1 uses the definition `\def~{\hphantom{0}}` to insert invisible spaces into columns of the table; see the source code for this document.

Very often tables containing results of Monte Carlo simulations use more digits than can be justified by the size of the simulation, and space can be gained and clarity improved by appropriate rounding. Standard errors or some other measure of precision should be given for Monte Carlo results. Often it suffices to give a phrase such as ‘The largest standard error for the results in column 2 is 0.01.’ in the caption to the table.

4.7. *Captions to figures and tables*

The caption to a figure should contain descriptions of lines and symbols used, but these should not be duplicated in the text, which should give the interpretation of the figure. The figure should not contain an inset. Verify that the caption and the graph agree, that every line and symbol is described correctly and that all lines or symbols in the graph are described in the caption.

Any abbreviations used in the body of a table should be explained in a footnote to it.

Figure captions always end with a full stop. The last sentence of a table title does not have a full stop.

4.8. *References*

References in the text should follow the current style used in *Biometrika*. It is preferable to use BibTeX if possible, as in this guide. In citing references use ‘First author et al.’ if there are three or more authors. The list of references at the end should correspond to those in the text, and be in exactly current *Biometrika* form.

References to books should be to the latest edition; a page, section or chapter number is nearly always necessary. References to books of papers should include title of book, editor(s), first and final page numbers of paper, where published and publisher.

Complete lists of authors and editors should be given; in exceptional cases they may be abbreviated at the discretion of the editor.

PhD theses, unpublished reports and articles can be referred to in the text, using a phrase such as ‘as shown in a 2009 Euphoric State University PhD thesis by M. Zapp (arXiv:1234.5678)’ or ‘the proofs may be found in an unpublished 2003 technical report available from the first author’, but should not be included in the References except where they have been accepted for publication (Berrendero, 2015). URLs should be avoided as they become obsolete quickly and it is preferable to refer to the authors and

institutions. Technical details for published papers should be prepared as supplementary material, so that they become permanently available. Likewise software should be submitted as supplementary material; of course it should be adequately documented, e.g., by including a README file to accompany `R` code.

Cox (1972) is an example of an active citation, and an example of a passive citation is (Heard et al., 2006). The abbreviations for their journals should be noted.

4.9. *Theorem-like environments*

Biometrika does not use LaTeX list environments such as `itemise`, `description`, or `enumerate`. In this subsection we illustrate the use of theorem-like environments.

DEFINITION 1 (OPTIONAL ARGUMENT). *This is a definition.*

Assumption 1 (*Another optional argument*). This is an assumption.

PROPOSITION 1. *This is a proposition.*

LEMMA 1. *This lemma precedes a theorem.*

Proof. This is a proof of Lemma 1. Perhaps it should be placed in the Appendix. □

THEOREM 1. *This is a theorem.*

Some text before we give the proof.

Proof of Theorem 1. The proof should be here. □

Example 1. This is an example.

Some text before the next theorem.

THEOREM 2 (OPTIONAL ARGUMENT). *Another important result.*

COROLLARY 1. *This is a corollary.*

Remark 1. This is a remark.

Step 1. This is a step.

Condition 1. This is a condition.

Property 1. This is a property.

Restriction 1. This is a restriction.

Algorithm 1. Finally, here is an algorithm.

Algorithm 1. A simple algorithm

Set $s = 0$
For $i = 1$ to $i = n$
  Set $t = 0$
  For $j = 1$ to $j = i$
    $t \leftarrow t + x_{ij}$
  $s \leftarrow s + t$
Output s

5. DISCUSSION

This is the concluding part of the paper. It is only needed if it contains new material. It should not repeat the summary or reiterate the contents of the paper.

Acknowledgement

Acknowledgements should appear after the body of the paper but before any appendices and be as brief as possible subject to politeness. Information, such as contract numbers, of no interest to readers, must be excluded.

Supplementary material

Further material such as technical details, extended proofs, code, or additional simulations, figures and examples may appear online, and should be briefly mentioned as Supplementary Material where appropriate. Please submit any such content as a PDF file along with your paper, entitled 'Supplementary material for Title-of-paper'. After the acknowledgements, include a section 'Supplementary material' in your paper, with the sentence 'Supplementary material available at *Biometrika* online includes . . .', giving a brief indication of what is available. However it should be possible to read and understand the paper without reading the supplementary material.

Further instructions will be given when a paper is accepted.

Appendix 1

General

Any appendices appear after the acknowledgement but before the references, and have titles. If there is more than one appendix, then they are numbered, as here.

Theorem A1. *This is a rather dull theorem:*

$$a + b = b + a; \tag{A1}$$

a little equation like this should only be displayed and labelled if it is referred to elsewhere.

Appendix 2

Technical details

Often the appendices contain technical details of the main results.

Theorem B1. *This is another theorem.*

Appendix 3

Often the appendices contain technical details of the main results:

$$a + b = c. \tag{C1}$$

Remark C1. This is a remark concerning equations (A1) and (C1).

References

Berrendero, J. R., Cuevas, A. & Torrecilla, J. L. (2015). On the use of reproducing kernel Hilbert spaces in functional classification. *arXiv*: 1507.04398v3.

Cleveland, W. S. (1993). *Vizualizing Data.* Summit: Hobart Press.

Cleveland, W. S. (1994). *The Elements of Graphing Data.* Summit: Hobart Press, revised ed.

Cox, D. R. (1972). Regression models and life tables (with Discussion). *J. R. Statist. Soc.* B **34**, 187–220.

Fan, J. & Peng, H. (2004). Nonconcave penalized likelihood with a diverging number of parameters. *Ann. Statist.* **32**, 928–61.

Heard, N. A., Holmes, C. C. & Stephens, D. A. (2006). A quantitative study of gene regulation involved in the immune response of Anopheline mosquitoes: An application of Bayesian hierarchical clustering of curves. *J. Am. Statist. Assoc.* **101**, 18–29.

R DEVELOPMENT CORE TEAM (2018). *R: A Language and Environment for Statistical Computing*. Vienna, Austria: R Foundation for Statistical Computing. ISBN 3-900051-07-0, http://www.R-project.org.
TUFTE, E. R. (1983). *The Visual Display of Quantitative Information*. Cheshire: Graphics Press.

[*Received on* 2 *January* 2017. *Editorial decision on* 1 *April* 2017]
