# Supplementary material for: Statistical properties of sketching algorithms
Source: Biometrika. Author manuscript; Available in PMC 2022 Feb 4. (PMC7612324; doi:10.1093/biomet/asaa062)
Supplement: Supplementary Material [file EMS140909-supplement-Supplementary_Material.zip › sketching_biometrika.zip!/RSSGraphs.pdf]

The Royal Statistical Society

How to present information in graphs and diagrams

Introduction

The Examinations Board has been greatly concerned at the extremely poor quality of graphs and diagrams submitted in recent years by candidates at all levels of the Society's examinations. These notes have been prepared in an attempt to give guidance to candidates; it may be expected that questions including the drawing of graphs and diagrams will feature in future examination papers perhaps more frequently than in the past. These notes are written in the context of representing economic data but are of general applicability. It is also hoped that they may be of value beyond the context of the Society's examinations.

Graphs and diagrams can, and usually do, serve two different functions. They can be useful analytical tools for professional statisticians, and they can be ways of displaying data to the public at large. The former include Normal probability plots, boxplots (box-and-whisker diagrams) and correlograms, and it can be expected that statisticians will be able to use them when appropriate and understand them without difficulty. However, it is also an important part of the work of statisticians that we should be able to present data in a coherent and meaningful manner to non-statisticians, and there are a great many methods of doing so. One can hardly find an issue of a serious newspaper that does not include several graphs. A single such picture conveys – or can convey – the important features of the data more vividly and memorably than columns of data.

It is unfortunate that many published graphs are inadequate, misleading, or in some cases incorrect. Holders of the Society's qualifications should know how to prepare good and useful graphs for presenting data to the general public, and should also be aware of errors and fallacies that all too readily arise in graphs prepared by others.

Titles, Labels and Footnotes

In principle, every graph should be self-contained, in that it should have a heading stating just what is being shown. Some details may be relegated to footnotes, or even the accompanying text, but the main features of the data should be given in the heading. The source of the data should be given, usually immediately below the graph. This enables the reader to assess the reliability of the data, to pursue apparent (or real) problems arising from them, and to clarify definitions if required. If the data come from a published source, it is helpful to give table or page numbers.

Footnotes should explain any points that might be unclear. A heading of "Gross Domestic Product, 1986 – 1996, £M" would require elaboration in a footnote to the effect that (for example, if appropriate) the data refer to the United Kingdom and are at constant 1990 factor cost.

Every graph and diagram should make immediately clear, in the heading, axis labels or footnote, what is being represented, the units and scale of measurement, the geographical coverage, the time or time period to which it relates, and the source of the data.

The axes of graphs should be labelled, even if the information is also given in the heading or footnote, eg the horizontal axis might be labelled "Year" and the vertical axis "£m", if appropriate.

Isotypes/pictographs

To attract and hold the interest of casual readers, one may decide to show changing annual consumption of wine, for example, by using bottles for each year. If each dimension of the bottle is in proportion to consumption, the volume of the bottle will be in proportion to the cube of consumption with alarming exaggerative effect, so this method of depicting data is to be avoided. It is much preferable to give different numbers of bottles of constant shape and size for each year's consumption, the numbers of bottles being in proportion to consumption so that each bottle represents a constant, and stated, quantity of wine. This is called the method of isotypes or pictographs.

Axes which do not start at zero

If either axis does not start from zero, this should be emphasised. A small zig-zag can be inserted in the axis between the origin and the first scale point. Any other breaks in the axis should be similarly marked. The least indication which is acceptable is a small gap in the axis. The most satisfactory indication, particularly in time charts, is a pair of wavy lines shortly above the time axis, suggesting that a strip has been torn out of the chart – as in a sense it has. Most official United Kingdom government publications ignore the need to mark axes which do not start from zero, as do most graphs in newspapers, thereby greatly diminishing their value. Without such an indication it will appear to readers that the distance of the graph from the horizontal axis is proportional to the data being displayed; the impression given by the graph will greatly exaggerate changes over time unless it is made abundantly clear that the vertical scale does not start from zero. Professional statisticians should not make this mistake.

Time Charts

In time charts, data relating to an entire quarter or year (eg Gross Domestic Product) are usually represented by a single point. The chart is thus technically complete if it consists of a set of such points at (usually) equal horizontal intervals. However, it will be much clearer if consecutive points are joined by straight lines, and this is usually done. Beginners sometimes try to join up the points by would-be smooth curves in a meaningless and unacceptable manner. (Statisticians may well superimpose a moving average or regression trend, which will not in general pass through all, or indeed any, of the actual observed points.)

Log Time Charts

Log time charts, in which equal vertical intervals indicate equal proportional differences (rather than equal absolute differences as with conventional scales), are a useful way of displaying and analysing some data, especially economic and financial statistics. These graphs can be confusing to lay people; this confusion should be minimised by clearly labelling the diagram as logarithmic and marking sufficient scale points on the vertical axis, which cannot start from zero. Such graphs should not be used if the readership is unlikely to understand them.

Scatter Diagrams

A scatter diagram marks with a dot or a small cross each bivariate observation (x_i, y_i). (Time charts are special cases of scatter diagrams.) If the number of observations is not large, it may be worth considering whether or not to label each point on the diagram. For data relating to a dozen standard regions, for example, where x is mean weekly household income and y is mean expenditure on motoring, it will be of interest to note which point relates to Greater London. By way of a further example, if the observations relate to Gross Domestic Product and to Imports, 1976 – 1996, not only can the points be labelled, but also successive observations can be joined by straight lines (an extension to two variables of the method used in time charts which is possible because the observations have occurred consecutively).

Histograms

Histograms are an extremely useful way of depicting data. Ideally the class intervals should be equal, and few special problems arise in such cases.

It is, however, sometimes necessary to draw histograms where the class intervals are unequal. It is essential that the *areas* of the different sections of a histogram should be made proportional to the frequencies being displayed, by making their heights proportional to the frequencies divided by the breadths of the class intervals. With unequal intervals it is useless to label any vertical axis "frequency density" or some such, since this will not be necessary for professional readers and will not be understood by the general public. Instead, an area scale should always be presented, of the form "one square centimetre = 100 households", with the square centimetre being shown as such on the histogram. One can often usefully give the actual frequencies in small circles in or above the rectangles of the histogram.

Histograms are often used to display distributions of incomes or ages, where the top interval is open-ended. There is no invariable rule as to how or whether to depict such intervals on the histogram but the problem cannot be merely ignored. Particularly when the class intervals are equal, there may be a natural approximate limit which can be used, though an explanatory footnote is necessary. In other contexts such as income distributions, it may often be best not

to attempt to show open-ended observations in the histogram but to explain the position in a footnote. In such cases it may be worth considering whether to use the area scale to construct, and present in the footnote, a block of the size appropriate to the relevant number of observations.

Bar charts

It is useful to distinguish between histograms, which display grouped data, and bar charts, which show frequencies of nominal or ordinal data. In a histogram, the bars should not be separated by blank spaces. In both histograms and bar charts, the number of observations in each category should be shown unless this is immediately evident from a vertical scale.

Conclusion

This short note can include only a few of the many points which examination candidates and others should bear in mind. Candidates are not expected to have the skills of professional draughtsmen, but should be able to draw neat, tidy, correct, suitable and helpful diagrams.

Further reading:

Darrell Huff, "How to Lie with Statistics", Penguin 1991 (or earlier editions).

Edward R Tufte, "The Visual Display of Quantitative Information", Graphics Press USA 1983.

Myra Chapman with Basil Mahon, "Plain Figures", Stationery Office 1997 (or earlier edition).

William S Cleveland, "Visualizing Data", Hobart Press USA 1993.

February 1998

Frank Oliver
University of Exeter

On behalf of the Examinations Board,
Royal Statistical Society

©RSS 1998

This material may be freely copied for teaching purposes provided its source is acknowledged. It may not be copied for any form of profit.
